# A Catalytic Osmium Redox Couple Collapses Cancer Redox Balance

**DOI:** 10.1002/advs.75576

**Published:** 2026-05-08

**Authors:** Wan‐Qiong Huang, Tao Huang, Yiming Hao, Gui‐Feng Huang, Yi‐Lang Yan, Xin Fang, Ying Chen, Xiao‐Long Wei, Wai‐Lun Man, Wen‐Xiu Ni

**Affiliations:** ^1^ Department of Pathology Cancer Hospital of Shantou University Medical College Shantou Guangdong P. R. China; ^2^ Department of Medicinal Chemistry Shantou University Medical College Shantou Guangdong P. R. China; ^3^ Department of Chemistry Hong Kong Baptist University Kowloon, HKSAR P. R. China; ^4^ Department of Thyroid and Breast Surgery, Clinical Research Center The First Affiliated Hospital of Shantou University Medical College Shantou Guangdong P. R. China

**Keywords:** bioinorganic chemistry, immunochemotherapy, osmium, reactive oxygen species, redox catalysis

## Abstract

Developing agents that disrupt the redox balance of tumor cells by simultaneously increasing reactive oxygen species (ROS) and decreasing antioxidants offers a promising approach in chemotherapy. Here, we present an isolable, interconvertible osmium redox pair, *trans*‐[Os^III^(NHPPh_3_)(L)(4‐Me_2_Npy)]^+^ and *trans*‐[Os^IV^(NHPPh_3_)(L)(4‐Me_2_Npy)]^2^^+^ [**Os(III)** and **Os(IV)**]. This self‐sustaining redox cycle of Os(III) and Os(IV) exhibit dual‐mode catalytic activity, experimental results show that **Os(III)** catalyses Fenton‐like activation of H_2_O_2_ to produce hydroxyl radicals, whereas **Os(IV)** oxidizes GSH to GSSG, regenerating **Os(III)** within cells. This cycle disrupts cellular redox homeostasis, prompting apoptosis and ferroptosis, and displays features of immunogenic cell death. In vivo, both **Os(III)** and **Os(IV)** inhibit tumor growth with good tolerability and enhance antitumor immune responses. These findings position redox‐cycling metal complexes as a promising strategy to target cancer redox vulnerabilities, encouraging further exploration of combination therapies with immunotherapeutic agents.

## Graphical abstract

A stable **Os(III)/Os(IV)** redox couple is developed to disrupt the tumor cell redox balance by concurrently catalyzing ROS generation and GSH depletion. Osmium‐treated cells exhibit multiple cell death pathways, including apoptosis, ferroptosis, and immunogenic cell death. In vivo, the couple suppresses tumor growth and activates antitumor immunity with good tolerability.

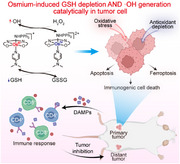

## 1 Introduction

Redox homeostasis is essential for cellular survival, growth, signal transmission, and other vital biological processes. This dynamic process involves a tightly regulated balance between multiple oxidative and antioxidant systems within cells, maintaining proper cellular functions. In cancer cells, increased metabolic activity raises reactive oxygen species (ROS) levels [1, 2, 3, 4], resulting in oxidative stress and ultimately causing oxidative cell death. To counteract this intrinsic oxidative stress, cancer cells develop a strong antioxidant defence system, primarily mediated by glutathione (GSH) [5, 6, 7]. Consequently, exploring agents that enhance oxidative stress or weaken intrinsic antioxidant defences has become a promising strategy in chemotherapy. The disruption of the redox balance in tumor cells can trigger lethal signalling cascades [8, 9, 10].

**FIGURE 1:**
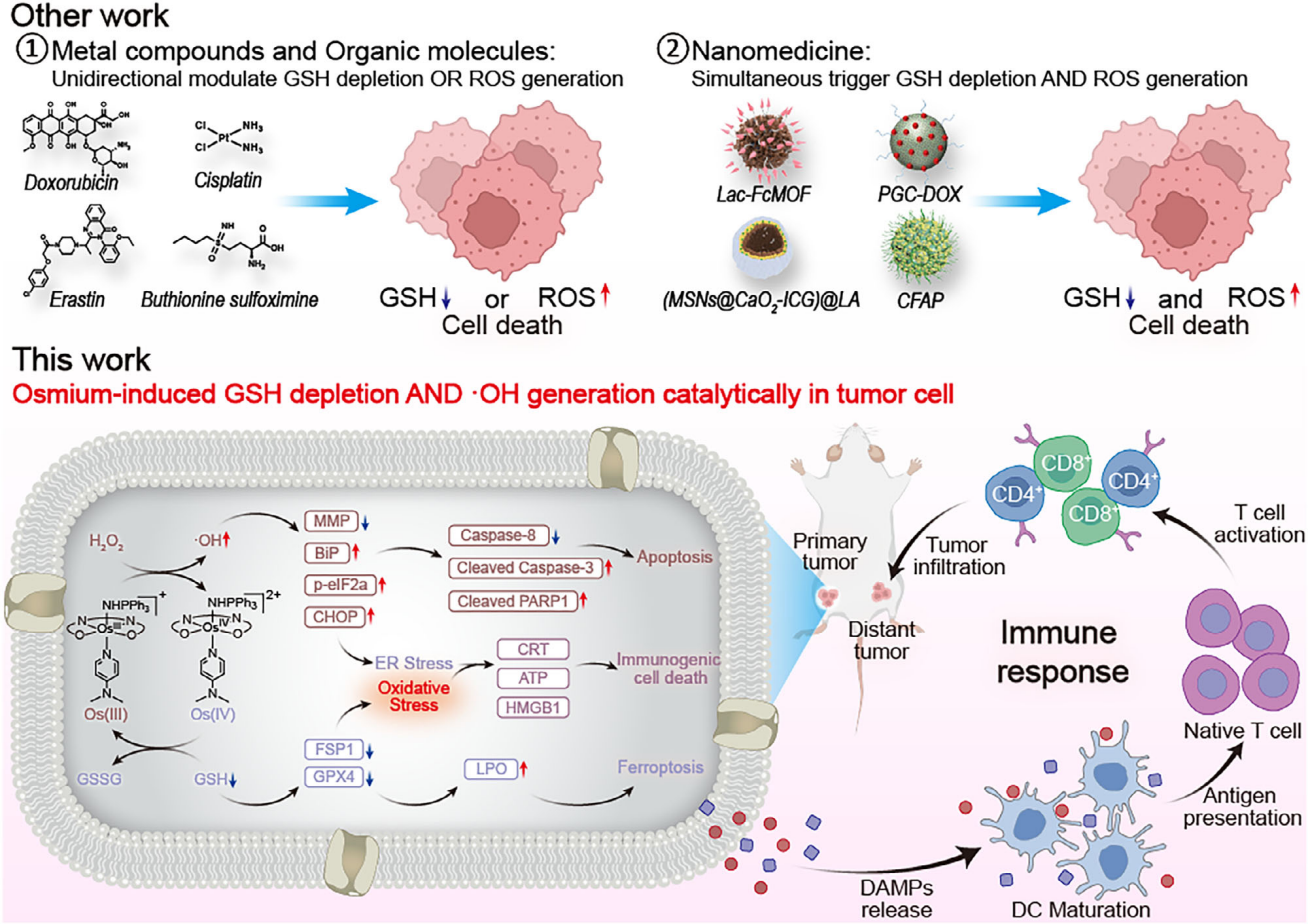
Unidirectional and bidirectional redox‐regulating agents. (Top Left): Metal complexes and organic molecules that solely deplete GSH or generate ROS [11]. (Top Right): Nanomedicines that can simultaneously trigger GSH depletion and ROS generation, but suffer from complex multicomponent architectures [14, 15, 16, 17]. This work: A proof‐of‐concept that an osmium redox couple concurrently depletes GSH and generates ^•^OH via a catalytic process within tumor cells. This disruption of redox homeostasis subsequently induces apoptosis, ferroptosis, and immunogenic cell death (ICD) in vitro. In vivo, the couple not only suppresses primary tumor growth but also effectively controls distant tumors through eliciting a systemic immune response.

Agents employing both unidirectional and bidirectional methods to alter the redox balance in tumor cells are known, as shown in Figure 1. Specifically, most existing agents adopt the unidirectional approach, either by increasing ROS production or by depleting GSH. Although these methods have shown promise in vitro, their effectiveness in complex biological systems is often limited by instability in the diverse conditions of tumor microenvironments [11, 12, 13]. Conversely, the bidirectional approach that synergistically enhances oxidative stress and depletes antioxidants offers a more promising multimodal approach for redox modulation. Recent notable examples include nanomaterial‐based complexes containing platinum, manganese, or copper [14, 15, 16, 17, 18, 19, 20, 21, 22, 23, 24]; however, these agents often display variable efficiency and substantial structural complexity, leading to inconsistent in vivo outcomes.

Molecular transition metal complexes, on the other hand, offer well‐defined structures and tunable reactivity. These complexes have been widely utilized as catalysts in numerous chemical transformations [25]. The catalytic activity of some complexes has been reviewed in living cells [26]. Notably, Sadler and others have reported a series of ruthenium(II) and osmium(II) organometallic complexes that demonstrate intracellular catalytic bifunctionality in regulating the redox balance [27, 28, 29, 30, 31, 32, 33]. Unfortunately, these complexes remain susceptible to speciation and thiol‐mediated deactivation. This challenge has prompted research into a new class of compounds that combine kinetic stability with reversible redox activity, allowing for catalytic amplification of ROS and concurrent GSH depletion under biologically relevant conditions.

In this work, we report a stable osmium redox couple, *trans*‐[Os^III^(NHPPh_3_)(L)(4‐Me_2_Npy)]^+^ and *trans*‐[Os^IV^(NHPPh_3_)(L)(4‐Me_2_Npy)]^2^^+^ (hereafter referred to as **Os(III)** and **Os(IV)**, respectively; L = *N,N′*‐bis(salicylidene)‐*o*‐cyclohexyldiamine dianion), which functions as a dual‐mode redox catalysis by interconverting between **Os(III)** and **Os(IV)** within cells. Our experimental results indicate that **Os(III)** catalyses Fenton‐like activation of H_2_O_2_ to produce hydroxyl radicals (^•^OH) (Equation (1), while **Os(IV)** oxidizes GSH to GSSG and is subsequently reduced back to **Os(III)** (Equation (2). This forms a self‐sustaining catalytic cycle that couples the induction of oxidative stress with the depletion of antioxidants.

(1) $$\mathrm{Os}\left(\mathrm{III}\right)+H_{2}O_{2}\to \mathrm{Os}\left(\mathrm{IV}\right){+}^{\cdot }\mathrm{OH}+OH^{-}$$

(2) $$\mathrm{Os}\left(\mathrm{IV}\right)+\mathrm{GSH}\to \mathrm{Os}\left(\mathrm{III}\right)1/2\;\mathrm{GSSG}+H^{+}$$

This bidirectional redox regulation disrupts the redox balance in tumor cells, inducing both apoptosis and ferroptosis, as well as eliciting hallmarks of immunogenic cell death in vitro. In vivo, both **Os(III)** and **Os(IV)** effectively suppress tumor growth while demonstrating more favourable tolerability than oxaliplatin. Our experimental findings are consistent with catalytic engagement within the tumor microenvironment and suggest a reduced reliance on single, resistance‐prone targets commonly associated with metallodrugs. These results establish metal‐based, bidirectional redox catalysis as a promising strategy for redox‐targeted cancer therapy. The simple, tunable scaffold and dual functionality of the **Os(III)**/**Os(IV)** couple overcome the inherent limitations of single‐mode redox‐modulators, providing insights for next‐generation therapeutics designed to exploit redox imbalance in cancer. (Figure 1)

## 2 Results and Discussion

### 2.1 Synthesis and Characterization of Redox‐Active Osmium Phosphinidine Complexes

**FIGURE 2:**
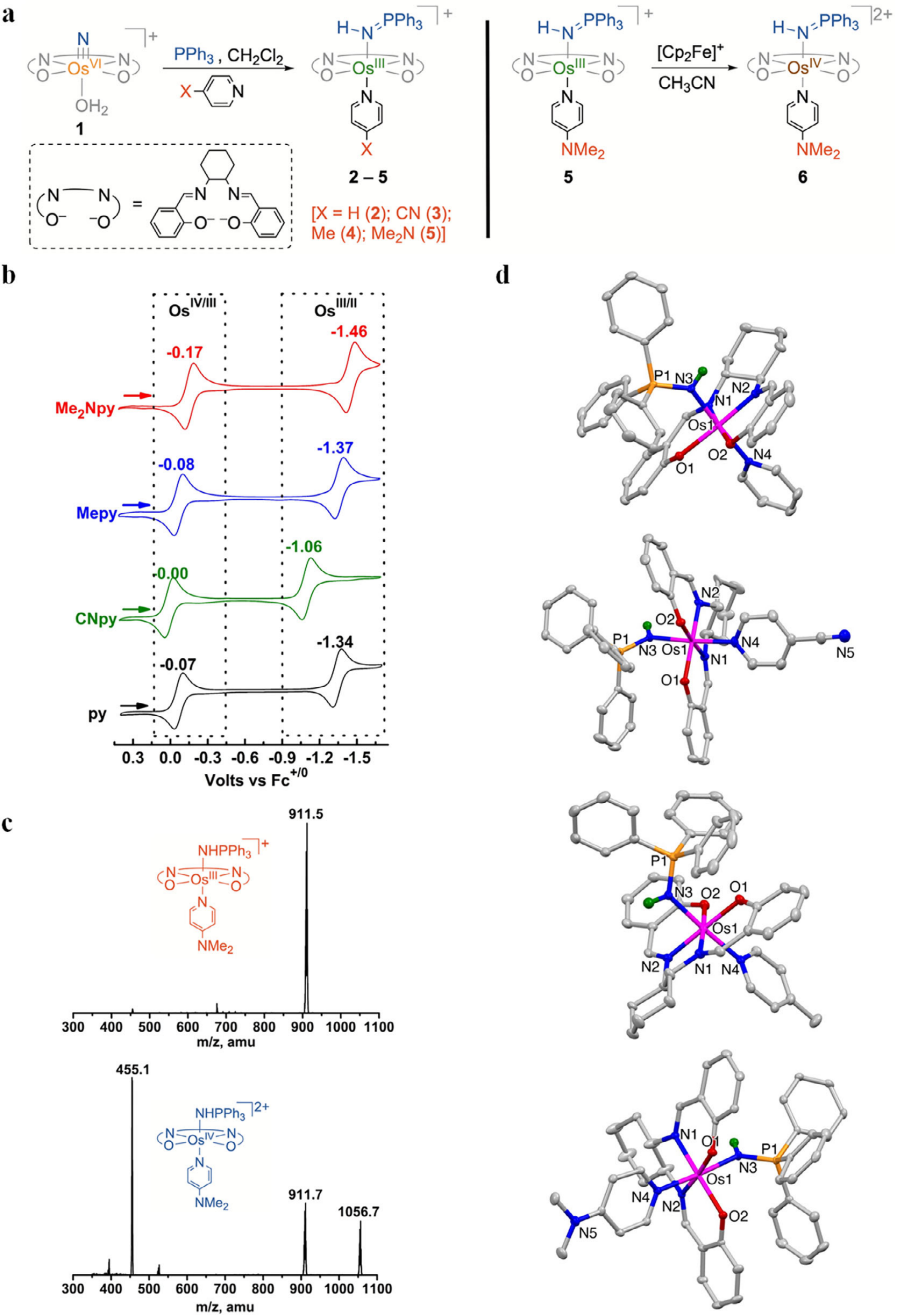
Synthesis and characterization of osmium phosphinidine complexes (**2–6**). (a) Synthetic scheme of **2**–**5** (left) and **6** (right). (b) CVs of **2**–**5** in 0.1 M [^n^Bu_4_N](PF_6_)/CH_3_CN, scan rate = 100 mV s^−1^. (c) ESI mass spectra of **5** (top) and **6** (bottom) in CH_3_CN. (d) Molecular structures of **2**–**5**; shown at 50% probability; hydrogen atoms (except N–H) are omitted for clarity.

We previously reported that [Os^III^(NHPPh_3_)(L)(pz)]^+^ (pz = pyrazine) is readily obtained by reacting [Os^VI^(N)(L)(OH_2_)]^+^ (**1**) with PPh_3_ in the presence of pz [34]. This complex shows a reversible Os(IV/III) couple at *E*_1/2_ = 0.00 V versus ferrocene in CH_3_CN, which lies within a physiologically relevant redox window. We sought to broaden and tune the Os(IV/III) potential systematically by replacing pz with *para*‐substituted pyridines (4‐Xpy). With a similar synthetic method, a series of osmium(III) complexes, [Os^III^(NHPPh_3_)(L)(4‐Xpy)]^+^ with X = H (**2**), CN (**3**), Me (**4**), and Me_2_N (**5**) has been synthesized (Figure 2a). Cyclic voltammetry (CV) analysis of **2–5** in CH_3_CN confirmed our strategy. The reversible Os(IV/III) couples span from 0.00 to −0.17 V versus ferrocene. The *E*_1/2_ shifts cathodically with increasing electron donation from X (Me_2_N > Me > H > CN; Figure 1b and Table S1). Importantly, the reversible Os(III/II) potentials of **2**–**5** are highly negative (*E*_1/2_ < −1 V vs. Cp_2_Fe^+^ in CH_3_CN), suggesting resistance to reduction to Os(II) under biological conditions. We also prepared the Os(IV) analogue, [Os^IV^(NHPPh_3_)(L)(4‐Me_2_Npy)]^2+^ (**6**), owing to the most cathodic Os(IV/III) potential and highest cytotoxicity (see below), for comparative biological studies. **6** was readily achieved by stoichiometric oxidation of **5** using ferrocenium hexafluorophosphate ([Cp_2_Fe]PF_6_) in CH_3_CN (Figure 2a).

All complexes (**2**–**6**) were well‐characterized. Infrared (IR) spectra display a weak ν(N–H) stretch at around 3340 cm^−1^, consistent with coordination of a neutral phosphinidine (NHPPh_3_) ligand (Figure S1 and Table S1). Electrospray ionization mass spectrometry (ESI‐MS) showed dominant parent ions for [Os^III^(NHPPh_3_)(L)(4‐Xpy)]^+^ (Figure S2). For example, **5** exhibits a single peak at mass‐to‐charge (*m/z*) 911.5 (Figure 2c). The oxidized analogue **6** shows two major ions at *m/z* = 455.1 and 1056.7 due to the dication [Os^IV^(NHPPh_3_)(L)(4‐Me_2_Npy)]^2+^ and the monocationic PF_6_^−^ adduct, respectively. These assignments are also validated by high‐resolution mass spectrometry (HR‐MS) (Figure S3). Single‐crystal X‐ray diffraction provided unambiguous structures for **2**–**6** (Figure 2d; Figure S4). They are isostructural, adopting distorted octahedral geometries with an equatorial salen plane. NHPPh_3_ and 4‐Xpy ligands are occupying the axial positions. Although the Os(IV/III) potentials are highly dependent on the para‐substituents of pyridine, the Os–NHPPh_3_ bond distances are similar across the Os(III) series (2.053–2.069 Å), indicating minimal trans influence from the para‐substituted pyridines in the solid state. Noteworthy, oxidation to Os(IV) induces contraction of the Os–NHPPh_3_ bond from 2.069 Å (**5**) to 2.027 Å (**6**), consistent with the higher oxidation state (Tables S2 and S3).

### 2.2 Initial Cytotoxicity Evaluation

Chemical stability was confirmed prior to cytotoxicity evaluation: each compound remained unchanged over a year in solid form. In solutions (DMSO and biological media), **2**–**5** were stable for at least 24 h by UV–vis spectroscopy (Figures S5 and S6). Cytotoxicity screening of complexes across multiple carcinoma cell lines and a normal cell line using the NBB assay (48 h exposure) demonstrated a clear trend (Table S4). In general, complexes **2**–**6** exhibit greater cytotoxicity toward cancer cells than normal cells. Amongst the osmium(III) series, complexes with stronger electron‐donating pyridines (Me_2_N > Me > H > CN; Figure S7), shows higher cytotoxicity, mirroring the Os(IV/III) redox potentials. This trend also aligns with the intracellular accumulation, determined by inductively coupled plasma mass spectrometry (ICP‐MS) (Figure S8a). For example, the most cytotoxic **5** (X = Me_2_N), exhibited an IC_50_ of 1.1 ± 0.1 µM. It also displays the highest cellular osmium accumulation (106.7 ± 18.5 ng Os per mg protein) in NCI‐H460 cells.

To understand the cell death mechanism, a series of in vitro experiments was conducted. **5** elicited the most significant increase in intracellular ROS (2.5‐fold over control, Figure S8b), consistent with redox activity under physiologically relevant conditions. Such oxidative stress compromises mitochondrial integrity [35], in line with the observed loss of mitochondrial membrane potential (2.1‐fold increase in the JC‐1 monomer/aggregate ratio by flow cytometry). Mitochondrial dysfunction was accompanied by reduced intracellular ATP [36] and increased extracellular ATP release (1.4‐fold over controls; Figure S8c, d), consistent with stress‐driven ATP flux. In addition, **5** triggered endoplasmic reticulum (ER) stress (upregulation of BiP and CHOP) and activated caspase‐dependent apoptosis pathways (upregulation of cleaved PARP1 and caspase‐3; Figure S9), aligning ROS‐driven signalling with apoptotic cell death. Flow cytometry corroborated increases in early (Annexin V^+^/PI^–^) and late (Annexin V^+^/PI^+^) apoptosis, totalling 41.7% (Figure S10). Because of its high cellular accumulation, robust ROS induction, and pronounced mitochondrial and ER perturbation, **5** was selected as the lead for in‐depth biological and mechanistic studies; its oxidized analogue, **6**, was included for comparison owing to its defined Os(III)/Os(IV) redox relationship.

### 2.3 Reversible Os(III)/Os(IV) Redox Cycling

**FIGURE 3:**
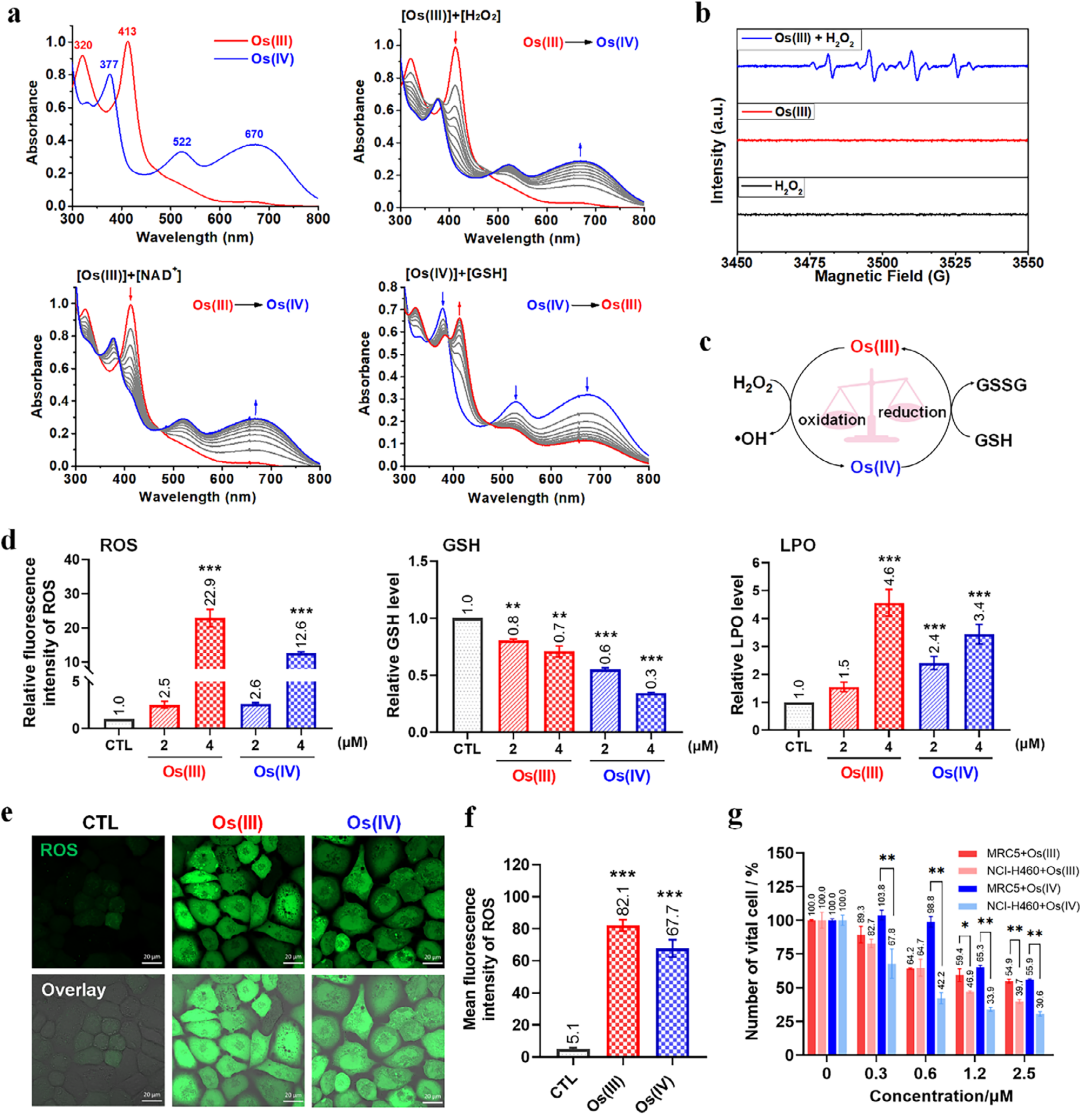
**Os(III)/Os(IV)** catalytic cycle induces intracellular oxidative stress and selective cancer cell death. (a) UV–vis spectroscopy in 50% aq. CH_3_CN. (Top Left): UV–vis spectra of pure **Os(III)** and **Os(IV)**; (Top Right): Reaction of 50 µM **Os(III)** with 5 mM H_2_O_2_ at 50 min intervals; total reaction time = 500 min; (Bottom Left): Reaction of 50 µM **Os(III)** with 5 mM NAD^+^ at 160 min intervals; total reaction time = 24 h; (Bottom Right): Reaction of 50 µM **Os(IV)** with 5 mM GSH at 160 min intervals; total reaction time = 24 h; (b) EPR spectra of the solution containing 200 µM **Os(III)** with 100 mM H_2_O_2_ (top), 200 µM **Os(III)** (middle), and 100 mM H_2_O_2_ (bottom) in the presence of DMPO as a spin trap. (c) Proposed catalytic cycle between **Os(III)** and **Os(IV)**. (d) Intracellular levels of ROS (6 h‐incubation), GSH (6 h‐incubation), and LPO (24 h‐incubation) in NCI‐H460 cells after treatment with 2 or 4 µM of **Os(III)** or **Os(IV)**. (e, f) Confocal fluorescence images and value statistics of intracellular ROS in NCI‐H460 cells after a 6 h‐treatment with 4 µM **Os(III)** or **Os(IV)** (Scale bar = 20 µm). (g) Cell viability of NCI‐H460 and MRC‐5 cells treated with **Os(III)** or **Os(IV)** for 48 h. Data are presented as mean±SD (n = 3), *p < 0.05, **p < 0.01, ***p < 0.001.

The above‐mentioned CVs of **5** and **6** (hereafter **Os(III)** and **Os(IV)**, respectively) display reversible Os(IV/III) behavior in CH_3_CN. We also investigated their interconvertible behaviour in 50% aqueous CH_3_CN using biologically relevant oxidants and reductants by UV–vis spectroscopy. Oxidation of **Os(III)** (50 µM) with H_2_O_2_ (5 mM) exhibited a spectral change to **Os(IV)**, confirmed by HR‐MS with the prominent ion at *m/z* 455.6496 (Figure S11a). At 8 h, conversion to approximately 77% **Os(IV)** was estimated based on the absorption peak at 670 nm (Figure 3a). Concurrently, we determined the diagnostic DMPO–OH quartet signal in electron paramagnetic resonance (EPR) spin‐trapping with DMPO, indicating the formation of hydroxyl radicals (^•^OH) (Figure 3b). Controls containing either **Os(III)** or H_2_O_2_ alone were EPR‐silent. This result is consistent with Fenton‐like activation of H_2_O_2_ by **Os(III)** (Equation (1). Again, oxidation of **Os(III)** to **Os(IV)** was also effective using NAD^+^, further supporting its feasibility under biologically relevant conditions (Figure 3a; Figure S11b). Reversibly, **Os(IV)** was easily reduced to **Os(III)** by GSH, as indicated by the decrease in **Os(IV)** absorptions at 522 and 670 nm and the increase of **Os(III)** absorption at 413 nm (Figure 3a). This transformation aligns with proton‐coupled electron transfer, which releases H^+^ and forms GSSG through GS^•^ dimerization (Equation (2). In control experiments, **Os(IV)** did not react with H_2_O_2_ or NAD^+,^ while **Os(III)** did not react with GSH. These findings demonstrate a chemically reversible **Os(III)**/**Os(IV)** cycle that catalytically couples ^•^OH production from H_2_O_2_ with GSH oxidation, which potentially supports continuous disruption of cellular redox balance (Figure 3c).

**Os(III)** and **Os(IV)** exhibit promising extracellular properties in regulating key redox balance components; in vitro potential was investigated. Treatment of NCI‐H460 cells with 4 µM **Os(III)** led to a 22.9‐fold increase in intracellular ROS and reduced GSH to 0.7‐fold (relative to control), accompanied by increased lipid peroxidation (LPO) (Figure 3d). Similarly, an increase of 12.6‐fold ROS and a decrease to 0.3‐fold GSH versus control were determined in 4 µM **Os(IV)**‐treated cells, supporting intracellular engagement of the **Os(III)**/**Os(IV)** couple to drive catalytic ROS amplification and GSH depletion. Confocal microscopy further revealed a significant increase in intracellular ROS levels following treatment (Figure 3e, f; Figure S12). Given these pro‐oxidant properties, we hypothesized preferential activity in cancer cells with high oxidative stress and elevated GSH pools [6, 37]; indeed, both **Os(III)** and **Os(IV)** were less cytotoxic to normal MRC5 cells (14.1 µg GSH per mg protein) than to NCI‐H460 cancer cells (62.5 µg GSH per mg protein) (Figure 3g; Figure S13), consistent with a favourable therapeutic window and selective engagement of tumor‐biased redox environments.

### 2.4 Cell Death Mechanisms

**FIGURE 4:**
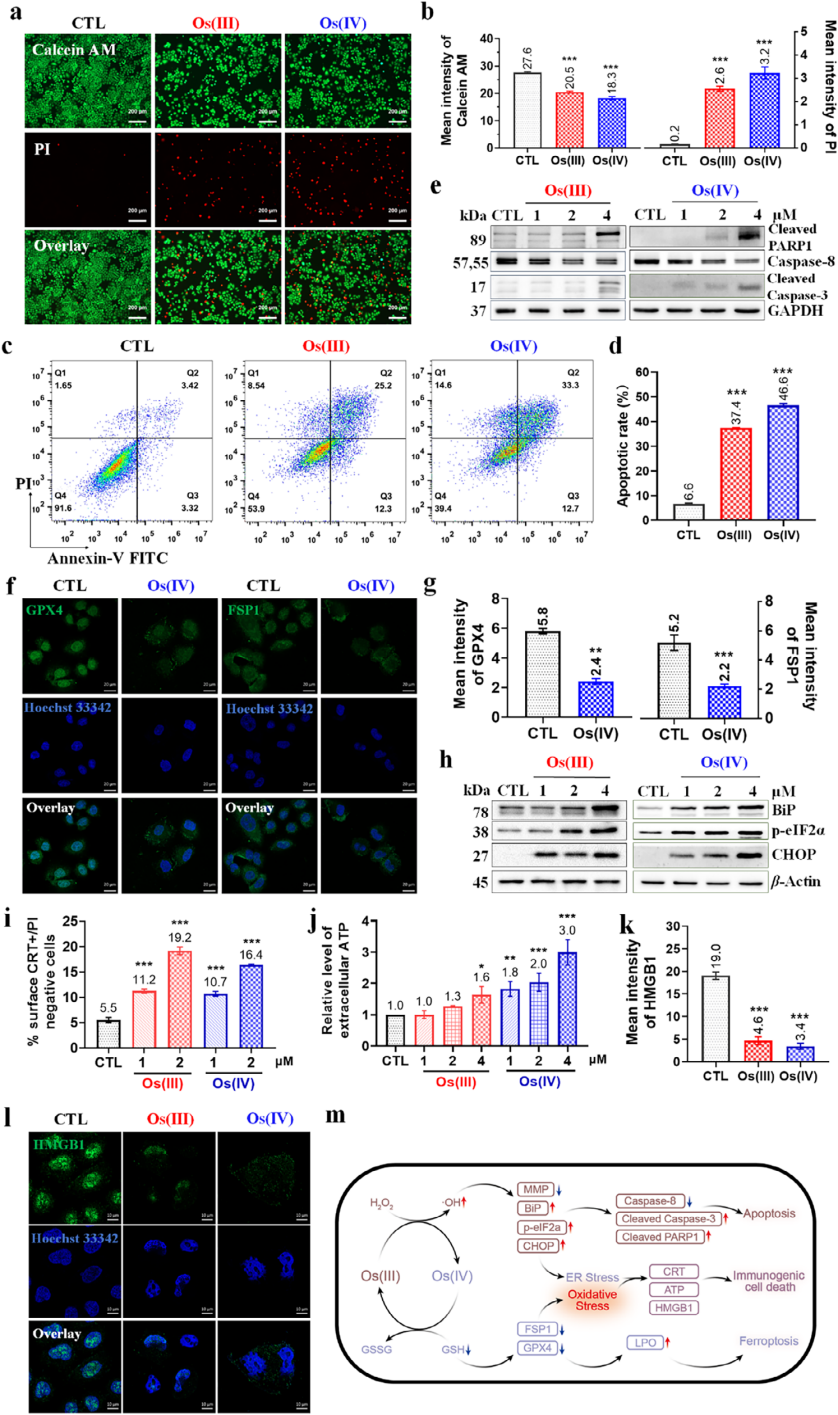
Cell death mechanisms and immunogenic responses induced by **Os(III)/Os(IV)** in NCI‐H460 cells after 24 h. (a) Live/dead staining of cells treated with **Os(III)/Os(IV)** (4 µM); Calcein AM (green) indicates live cells, PI (red) indicates dead cells (Scale bar = 200 µm). (b) Mean fluorescence intensity of Calcein AM and PI. (c, d) Flow cytometry analysis and corresponding quantifications analysis of apoptotic populations in cells following treatment with **Os(III)/Os(IV)** (4 µM). (e) Western blot analysis of apoptosis‐related proteins in cells exposed to **Os(III)/Os(IV)** (1, 2, and 4 µM). (f) Immunofluorescence detection of GPX4 and FSP1 (green channel, Ex = 488 nm) in cells treated with **Os(IV)** (4 µM), visualized by LSCM (Scale bar = 20 µm). (g) Mean fluorescence intensity of GPX4 and FSP1. (h) Representative immunoblot images of ER stress‐related proteins in cells treated with **Os(III)/Os(IV)** (1, 2, and 4 µM). (i) Flow cytometry quantification of cell surface CRT expression after **Os(III)/Os(IV)** treatment (1 and 2 µM). (j) ATP release in cell culture supernatants was measured after treatment with **Os(III)/Os(IV)** (1, 2, and 4 µM). (k, l) Confocal fluorescence imaging and corresponding quantifications analysis of HMGB1 release in cells, detected with a specific antibody after **Os(III)/Os(IV)** (4 µM) treatment (Scale bar = 10 µm). (m) Schematic illustration of the proposed mechanisms of **Os(III)/Os(IV)**‐induced cell death and immune activation. Data are presented as mean±SD (n = 3), *p < 0.05, **p < 0.01, ***p < 0.001.

Given the potent cytotoxicity and redox activity of **Os(III)** and **Os(IV)**, we profiled cell death pathways in NCI‐H460 cells. Both complexes caused pronounced loss of viability consistent with oxidative injury, reflected by decreased Calcein‐AM positive (live) cells and increased propidium iodide (PI) positive (dead) cells (Figure 4a, b; Figure S14). Annexin V/PI co‐staining showed substantial increases in early (AV^+^PI^−^) and late (AV^+^PI^+^) apoptotic populations (Figure 4c, d; Figure S15), corroborated by activation of apoptotic markers, including cleaved PARP1 and cleaved caspase‐3 (Figure 4e; Figure S16). Because depletion of intracellular GSH compromises GPX4 activity and favours ferroptosis [38, 39], we next assessed ferroptotic signatures. Consistent with Os‐induced GSH depletion and elevated LPO (Figure 3d), **Os(IV)** markedly suppressed GPX4 and FSP1, two key ferroptosis defence proteins, by western blot and immunofluorescence, whereas **Os(III)** showed no comparable suppression, indicating divergent contributions of the two oxidation states (Figure 4f, g; Figures S17–S19). Pathway‐specific inhibitors further supported these assignments [40]: the pan‐caspase inhibitor Z‐VAD‐FMK significantly rescued **Os(III)**‐treated cells, implicating apoptosis as the primary death mode, whereas **Os(IV)**‐induced death was attenuated by both Z‐VAD‐FMK and the ferroptosis inhibitor ferrostatin‐1, consistent with mixed caspase‐dependent and ferroptotic contributions (Figures S20, S21).

Both **Os(III)** and **Os(IV)** perturbed ER proteostasis, with CHOP upregulation, increased eIF2a phosphorylation, and altered BiP levels, indicative of an activated unfolded protein response (Figure 4h; Figure S22). Prolonged ER stress was associated with ER Ca^2^^+^ release and elevated cytosolic Ca^2^^+^, detected by increased Fluo‐4 AM fluorescence (Figures S23 and S24). These data indicate that dynamic **Os(III)**/**Os(IV)** redox cycling imposes oxidative stress beyond cellular tolerance, eliciting ER stress and Ca^2+^ dysregulation, mitochondrial injury, and activation of apoptosis (predominant for **Os(III)**) with a stronger ferroptotic component for **Os(IV)**. This multimodal cell‐death response underlies the efficient eradication of NCI‐H460 cancer cells.

### 2.5 Stimulation of Anti‐Tumor Immunity in Vitro

Given the immunomodulatory roles of ROS and ER stress [41, 42], and the interplay between regulated cell death (apoptosis and ferroptosis) and anti‐tumor immune activation [43, 44], we evaluated hallmarks of immunogenic cell death (ICD), which is characterized by emission of damage‐associated molecular patterns (DAMPs), including calreticulin (CRT) exposure, extracellular ATP release, and HMGB1 secretion, that recruit and activate of anti‐tumor immunity [45, 46, 47]. NCI‐H460 cells treated with **Os(III)** or **Os(IV)** (24 h) showed significant CRT translocation to the plasma membrane by flow cytometry (19.2% and 16.4% CRT‐positive cells) (Figure 4i). Meanwhile, we conducted a co‐localization experiment using the membrane‐specific probe Dil (a lipophilic carbocyanine dye) in combination with CRT immunofluorescence staining. As shown in Figure S25, treatment with **Os(III)** or **Os(IV)** resulted in a marked increase in CRT signal on the cell membrane, as evidenced by co‐localization signals in the overlay images. These findings indicate translocation of CRT to the membrane surface. Extracellular ATP increased 1.6‐fold for **Os(III)** and 3.0‐fold for **Os(IV)**, relative to control (Figure 4j), consistent with stress‐associated ATP efflux; confocal imaging revealed pronounced HMGB1 release, and ELISA quantified 8.0‐fold and 17.3‐fold elevations for **Os(III)** and **Os(IV)**, respectively, in extracellular HMGB1 (Figure 4k, l; Figures S26 and S27). These DAMPs emissions align mechanistically with the observed ER stress (CHOP upregulation and elF2a phosphorylation) and ROS induction, both known triggers of ICD. Thus, **Os(III)** and **Os(IV)** not only eliminate cancer cells via overlapping apoptotic and ferrototic pathways, but also elicit canonical ICD signals, potentially priming anti‐tumor immune responses. (Figure 4m)

### 2.6 Antitumor Efficacy and Immune System Activation In Vivo

Encouraged by the in vitro potency, we first assessed the antitumor efficacy by tail vein intravenous injection of complexes in BALB/c nude mice bearing subcutaneous NCI‐H460 xenografts (Figure S28a). By day 20, mean tumor volumes reached 783.9 ± 368.2 mm^3^ (vehicle group) and 427.6 ± 309.8 mm^3^ (oxaliplatin), whereas treatment with 5 mg/kg **Os(III)** or **Os(IV)** resulted in significantly smaller tumors of 261.0 ± 118.1 mm^3^ and 207.6 ± 139.9 mm^3^, respectively (Figure S28b). Final tumor weights corroborated robust growth suppression, with inhibition rates of 66% and 74% for **Os(III) and Os(IV)**, respectively, versus 37% for oxaliplatin (Figure S28c). Histology showed extensive necrosis (cellular fragmentation, nuclear dissolution, and chromatin condensation) in **Os(III)** and **Os(IV)** cohorts, contrasting with preserved architecture in controls (Figure S29). No significant body‐weight loss or histopathological abnormalities in heart, liver, spleen, lung, or kidney were observed, indicating a favourable safety profile (Figures S28d and S30).

**FIGURE 5:**
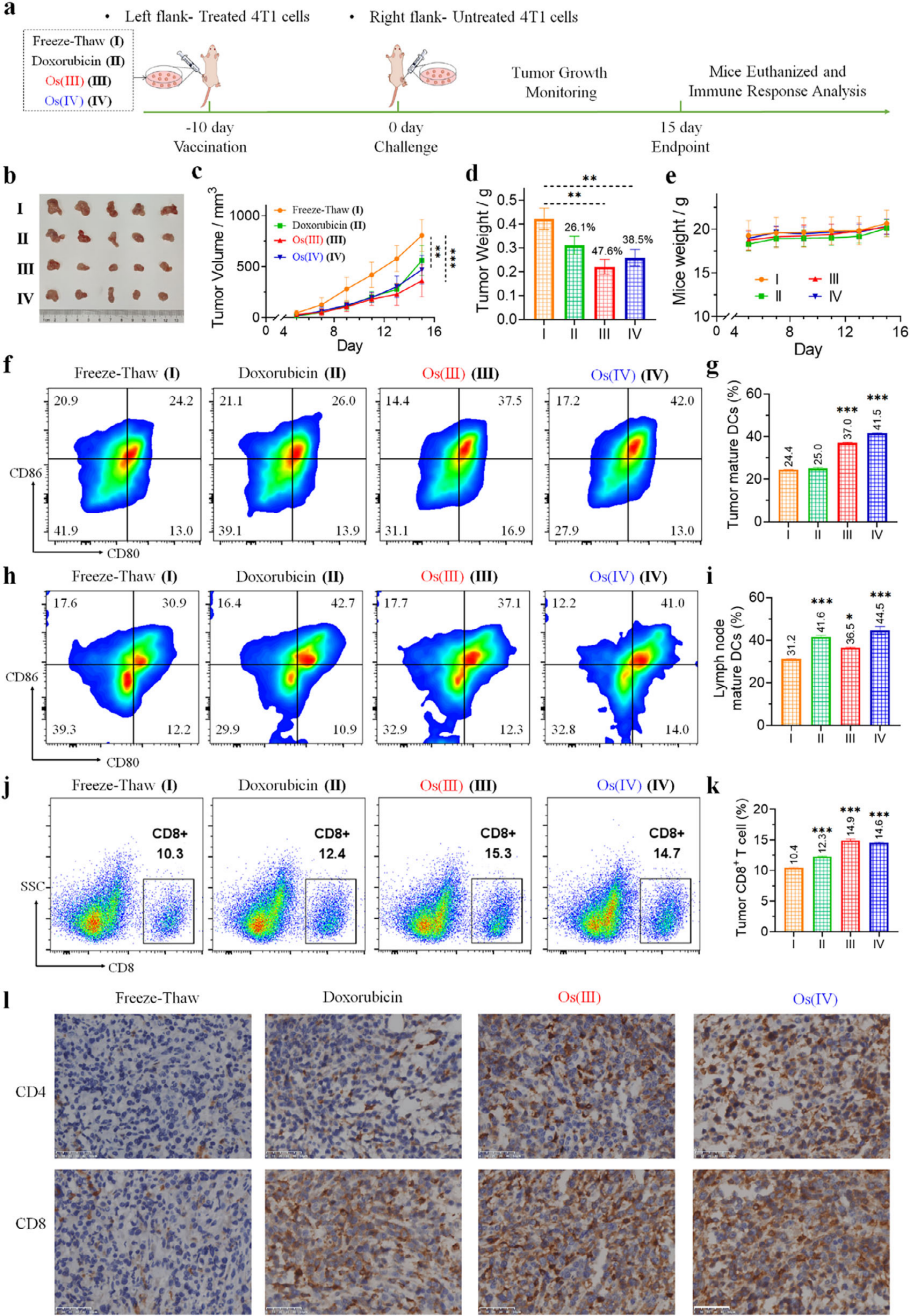
Evaluation of ICD immunogenicity in vivo by **Os(III)/Os(IV)**. (a) Schematic diagram of the therapeutic vaccination schedule in 4T1 tumor‐bearing BALB/c mice. Mice were randomly divided into four groups (*n*  = 5 per group): Freeze‐thaw (Negative control, Group I), Doxorubicin (30 µM, ICD‐positive control, Group II), **Os(III)** (10 µM, Group III), and **Os(IV)** (10 µM, Group IV).  The following analysis is based on the right‐sided tumors. (b) Image of isolated tumor tissues at day 15. (c) Growth curves of tumor volume. (d) Tumor weight at the endpoint. (e) Body weight of mice under different treatments. The proportion and quantitative analysis of CD80^+^CD86^+^ antigen‐presenting cells in the tumor tissues (f, g) and lymph nodes (h, i) at day 15. (j, k) The proportion of CD8^+^ cytotoxic T lymphocytes in the tumor tissues at day 15. (l) Immunohistochemistry of CD4^+^ and CD8^+^ T cells in tumor tissues (scale bar = 50 µm). Data are presented as mean±SD, *p < 0.05, **p < 0.01, ***p < 0.001.

To evaluate ICD‐linked immunogenicity in vivo [48], we conducted a prophylactic vaccination study using 4T1 cells in immunocompetent BALB/c mice (Figure 5a). Mice were vaccinated on the left flank with **Os(III)**‐ or **Os(IV)**‐treated 4T1 cells on day ‐10; freeze‐thawed necrotic and doxorubicin‐treated 4T1 cells served as negative and positive vaccine controls, respectively. Untreated 4T1 cells were implanted contralaterally on day 0. After 15 days, contralateral tumor growth was significantly attenuated in the **Os(III)** and **Os(IV)** vaccine groups, with inhibition rates of 47.6% and 38.5%, respectively–both surpassing doxorubicin at 26.1% (Figure 5b–d). Body weights remained stable across groups, indicating good tolerability (Figure 5e).

Immune profiling supported enhanced antitumor immunity. Flow cytometry showed increased dendritic‐cell maturation (CD80^+^CD86^+^) within tumors to 37.5% and 42.0% for **Os(III)** and (**Os(IV)**, respectively, compared to 26.0% and 24.2% in the positive and negative vaccine controls, respectively (Figure 5f, g), with parallel increases in draining lymph nodes (37.1% and 41.0%, for **Os(III)** and **Os(IV)**, respectively; Figure 5h, i). Tumor‐infiltrating lymphocytes analysis showed elevated CD8^+^ cytotoxic T‐cell frequencies from 10.3% (negative control) to 15.3% (**Os(III)**) and 14.7% (**Os(IV)**) (Figure 5j, k). Immunohistochemical analysis confirmed robust infiltration of both CD4^+^ helper and CD8^+^ cytotoxic T cells in treated tumors (Figure 5l) [49, 50].

Together, the prophylactic vaccination experiment indicates that **Os(III)**‐ and **Os(IV)**‐treated 4T1 cells can elicit protective antitumor immunity upon subsequent tumor challenge, as evidenced by significantly slower tumor growth in vaccinated mice compared with controls. These findings provide functional support that the DAMP‐based ICD signatures observed in vitro are accompanied by in vivo immunogenicity, consistent with the priming of the antitumor immune response capable of controlling the growth of live tumor cells. These findings establish a direct mechanistic link: Os‐induced ICD in tumor cells serves as the initiating trigger for dendritic cell maturation, which in turn drives T cell priming and intratumoral infiltration, ultimately leading to tumor growth inhibition.

**FIGURE 6:**
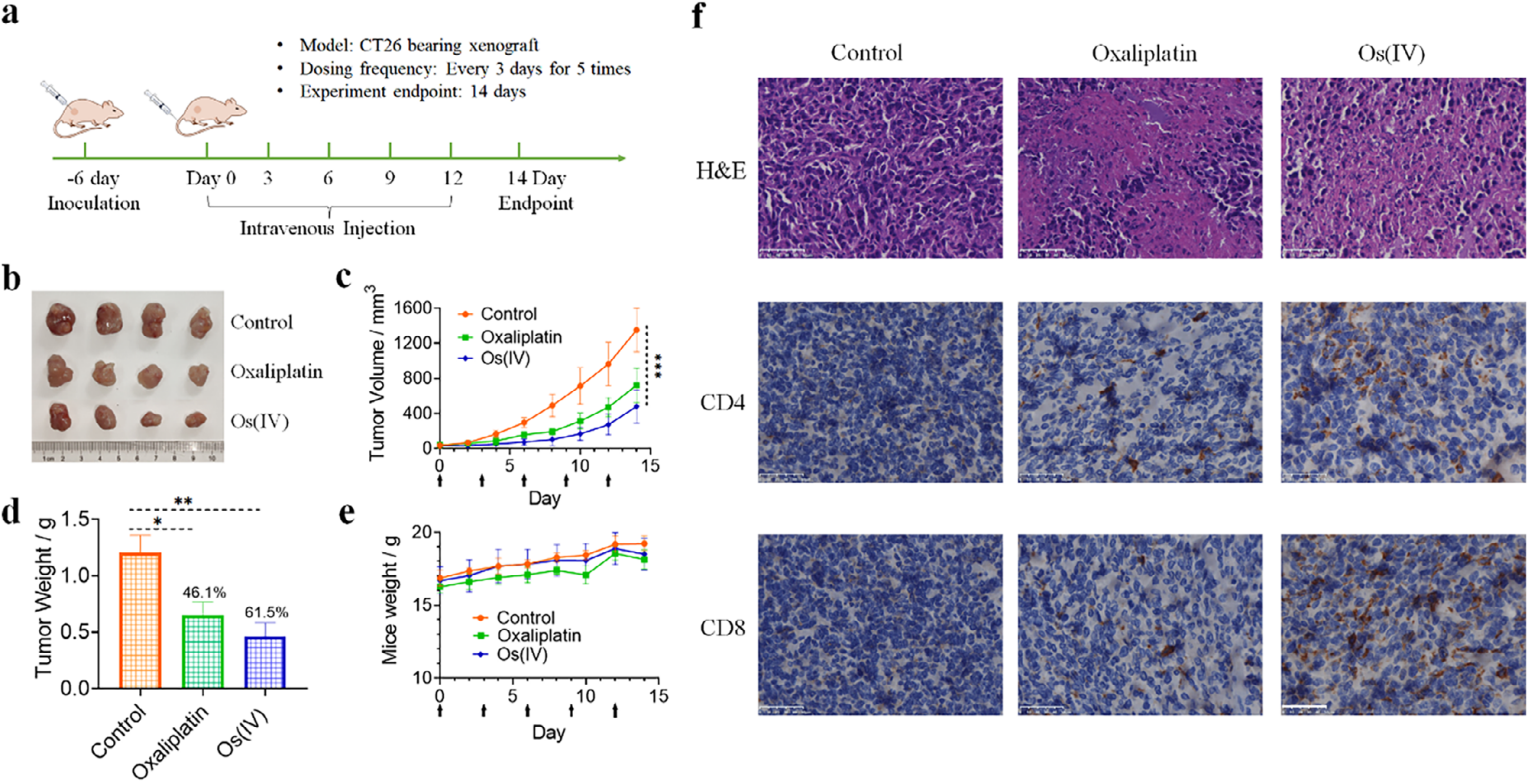
Antitumor effect and immune response analysis of **Os(IV)**. (a) Schematic of the treatment schedule in CT26 tumor‐bearing BALB/c mice. Mice receiving intravenous injections every 3 days were divided into different groups: control (10% PET), oxaliplatin (3 mg/kg), or **Os(IV)** (5 mg/kg). (b) Image of isolated tumor tissues at day 15. (c) Changes in relative tumor volume during therapy. (d) Weight of isolated tumors. (e) Body weight of mice receiving treatment. (f) H&E staining and immunohistochemistry of CD4^+^ and CD8^+^ T cells in tumor tissues (Scale bar = 50 µm). Data are presented as mean±SD, *p < 0.05, **p < 0.01, ***p < 0.001.

To further verify immune activation, we assessed the efficacy of **Os(IV)** using an immunocompetent BALB/c CT26 xenograft model (Figure 6a). Mice receiving intravenous injections every 3 days for 14 days were divided into different groups: (10% PET), oxaliplatin (3 mg/kg), or **Os(IV)** (5 mg/kg). By study end, mean tumor volumes of groups control, oxaliplatin and Os(IV) were determined to be 1351.6, 721.6, and 480.7 mm^3^, respectively. Endpoint tumor weights showed a 61.5% inhibition with **Os(IV)**, outperforming oxaliplatin's 46.1% (Figure 6b–d). Again, no significant body‐weight loss or histopathological abnormalities in heart, liver, spleen, lung, or kidney were observed, indicating good tolerability (Figure 6e; Figure S31). Histology revealed substantial tumor necrosis in treated groups, and immunohistochemical staining indicated significant infiltration of CD4^+^ helper and CD8^+^ cytotoxic T cells within the tumors (Figure 6f). Consistent with their respective immunomodulatory roles [51, 52, 53, 54], immunohistochemical analysis revealed that **Os(IV)** treatment markedly increased CD20^+^ B cell infiltration while reducing Foxp3^+^ Treg infiltration within the tumor microenvironment compared to control and oxaliplatin groups (Figure S32). Flow cytometry of the spleen further confirmed systemic immune activation, with CD4^+^ and CD8^+^ T‐cell populations increasing from 19.9% to 23.4% and from 8.6% to 10.4%, respectively (Figure S33). Further analysis revealed that **Os(IV)** treatment increased splenic CD80^+^/CD86^+^ DCs (from 15.3% to 22.7% vs. control, Figure S34), indicating enhanced DC maturation. These increases are consistent with ICD‐associated immune activation, in which DMAP emissions (CRT exposure, extracellular ATP release, HMGB1 secretion) promote DC maturation and T‐cell activation. Overall, these results demonstrate that **Os (IV)** exert strong antitumor effects in vivo with minimal toxicity, and promote ICD‐linked immune activation, including increased T‐cell infiltration and systemic T‐cell expansion.

Interestingly, the stronger tumor infiltration signal with **Os(IV)** suggests it may be more effective in promoting final T cell recruitment or survival within the TME. In addition, the relatively lower T cell percentage in the spleen for the **Os(IV)** group compared to Oxaliplatin could indicate different pharmacological profiles [51, 55, 56]. Together, **Os(IV)** effectively primes dendritic‐cell maturation, likely facilitating rapid T cell egress from the spleen and preferential homing to the tumor site. This interpretation is reinforced by the coordinated increase in both T and B cells within the tumor microenvironment, indicating that **Os(IV)** uniquely remodels the tumor immune landscape despite moderate systemic expansion.

Biodistribution analysis by ICP‐MS revealed that **Os(III)/(IV**) complexes are systematically distributed in CT26 and NCI‐H460 tumor‐bearing mice, with preferential accumulation in metabolic and immune organs (kidney and spleen), rather than selective enrichment in the tumor (Figure S35). We have evaluated the stability of the Os complex in red blood cells. As presented in Figure S36, it is worth noting that at the higher concentration tested (10 µM), slight hemolysis was observed at the 48‐hour time point, suggesting concentration‐dependent effects on erythrocyte stability at prolonged exposure.

Despite their pronounced immune‐mediated antitumor efficacy, **Os(III)/(IV)** complexes exhibit only moderate tumor selectivity and tend to accumulate in the kidneys and spleen. The mild hemolysis observed at higher concentrations further underscores the importance of dosage optimization. Future research will focus on developing nanomedicine‐based strategies to enhance tumor‐specific delivery, while parallel structural optimization efforts aim to reduce hemolytic effects without compromising immunomodulatory activity. These improvements should enhance the therapeutic index and clinical translation potential of osmium‐based complexes.

## 3 Conclusions

We demonstrate that kinetically stable osmium complexes can engage in a reversible Os(IV/III) redox cycle that actively reshapes intracellular redox homeostasis. This cycle orchestrates simultaneous ROS generation and GSH depletion, synergistically inducing apoptosis and ferroptosis characteristics, alongside activation of ER stress‐associated pathways indicative of immunogenic cell death. These cellular processes are associated with the maturation of dendritic cells and increased infiltration of intratumoral CD4^+^ and CD8^+^ T cells, contributing to pronounced tumor suppression with favourable tolerability in vivo. The kinetic inertness, adaptable ligand environment, and tunable redox properties of our osmium complexes afford precise manipulation and investigation of this chemical cycle both in cellular and animal models.

Our results introduce a design strategy that leverages mid‐ to late‐transition‐metal pairs with tunable ligands to coordinate ROS generation and GSH oxidation in a controlled fashion, thereby reprogramming cellular redox systems to amplify antitumor immunity responses. By varying the pyridine ligand, it is possible to fine‐tune both the redox potential and reaction rate, enabling this approach to be extended to ruthenium analoges, and to other transition‐metal platforms. This tunability paves the way for rational optimization and combination therapies, including immune checkpoint blockade and other interventions targeting cellular redox. While our work establishes the chemical cycle and its biological implications, further genetic and kinetic studies are warranted to confirm mechanisms, delineate operational ranges in cells, and inform development toward clinically applicable metal agents and combination treatments.

Future studies will pursue definitive functional validation of ICD in therapeutic settings by: (i)conducting survival‐based tumor rechallenge in mice that achieve tumor control to assess the durability of immunological memory; and (ii) testing combinations with immune checkpoint inhibitors (e.g., anti‐PD‐1/PD‐L1) to evaluate potential synergy. These experiments will more rigorously establish the translational potential of osmium complexes as immunochemotherapeutic agents.

## 4 Experimental Section

### 4.1 General Synthesis of [Os^III^(NHPPh_3_)(L)(4‐Xpy)](PF_6_)

PPh_3_ (28 mg, 0.11 mmol) was added to a mixture of [Os^VI^(N)(L)(OH_2_)](PF_6_) (69 mg, 0.1 mmol) and excess 4‐Xpy (3.00 mmol) in CH_2_Cl_2_ (50 mL). The resulting solution was stirred at room temperature for 24 h to afford a brown solution, which was concentrated and loaded onto a silica gel (20 × 2 cm). The column was first flushed with CH_2_Cl_2_. Then, the brown fraction was eluted with a mixture of CH_2_Cl_2_/acetone (30:1, v/v). Evaporation of the volatiles yielded an analytically pure brown microcrystalline solid. Single crystals suitable for X‐ray analysis were obtained by slow diffusion of diethyl ether into the CH_3_CN solution at room temperature.

*[Os^III^(NHPPh_3_)(L)(py)](PF_6_)* **2(PF_6_)**. Yield: 45%. IR (KBr in cm^−1^): ν(N–H), 3339; ν(C = N), 1603; ν(P = N), 1147; ν(P–F), 832. Anal. calcd. (found) for C_43_H_41_F_6_N_4_O_2_OsP_2_: C, 51.04 (51.13); H, 4.08 (4.19); N, 5.54 (5.48). ESI/MS in CH_3_CN: m/z: 868 (M^+^). HRMS: m/z Calcd. for C_43_H_41_F_6_N_4_O_2_OsP_2_ ([M]^+^): 868.2576, found: 868.2575.

*[Os^III^(NHPPh_3_)(L)(4‐CNpy)](PF_6_)* **3(PF_6_)**. Yield: 40%. IR (KBr in cm^−1^): ν(N–H), 3341; ν(C≡N), 2240; ν(C = N), 1601; ν(P = N), 1148; ν(P–F), 834. Anal. calcd. (found) for C_44_H_40_F_6_N_5_O_2_OsP_2_: C, 50.96 (50.51); H, 3.89 (4.25); N, 6.75. ESI/MS in CH_3_CN: m/z: 893 (M^+^). HRMS: m/z Calcd. for C_44_H_40_F_6_N_5_O_2_OsP_2_ ([M]^+^): 893.2529, found: 893.2533.

*[Os^III^(NHPPh_3_)(L)(4‐Mepy)](PF_6_)* **4(PF_6_)**. Yield: 50%. IR (KBr in cm^−1^): ν(N–H), 3340; ν(C = N), 1601; ν(P = N), 1148; ν(P–F), 832. Anal. calcd. (found) for C_44_H_43_F_6_N_4_O_2_OsP_2_: C, 51.51 (51.74); H, 4.22 (4.27); N, 5.46 (5.31). ESI/MS in CH_3_CN: m/z: 882.6 (M^+^). HRMS: m/z Calcd. for C_44_H_43_F_6_N_4_O_2_OsP_2_ ([M]^+^): 882.2733, found: 882.2734.

*[Os^III^(NHPPh_3_)(L)(4‐Me_2_Npy)](PF_6_)* **5(PF_6_)**. Yield: 48%. IR (KBr in cm^−1^): ν(N–H), 3335; ν(C = N), 1601; ν(P = N), 1150; ν(P–F), 833. Anal. calcd. (found) for C_45_H_46_F_6_N_5_O_2_OsP_2_: C, 51.23 (51.30); H, 4.39 (4.40); N, 6.64 (6.45). ESI/MS in CH_3_CN: m/z: 911 (M^+^). HRMS: m/z Calcd. for C_45_H_46_F_6_N_5_O_2_OsP_2_ ([M‐PF_6_]^+^): 911.2998, found: 911.3003. UV–vis in 50% aqueous CH_3_CN, l_max_ [nm] (e[mol^−1^dm^3^cm^−1^]): 256 (32000), 321 (18360), 413 (20100), 526 (2675), 660 (540).

### 4.2 Synthesis of [Os^IV^(NHPPh_3_)(L)(4‐Me_2_Npy)](PF_6_)_2_ **6(PF_6_)_2_**

Ferrocenium hexafluorophosphate (70 mg, 0.21 mmol) was added to **5(PF_6_)** (260 mg, 0.25 mmol) in 30 mL CH_3_CN, and the solution was stirred at room temperature for 15 min. The resulting dark blue solution was evaporated to dryness and washed with diethyl ether (3 × 10 mL). The dark blue solid was redissolved into CH_2_Cl_2_ and loaded onto a silica gel column (25 × 2.5 cm). The column was initially eluted with CH_2_Cl_2_/acetone (30:1 v/v), followed by CH_2_Cl_2_/acetone (10:1 v/v). The dark blue fraction was collected and evaporated to dryness, yielding a dark blue solid. Yield: (137 mg, 46%). Anal. calcd. (found) for C_45_H_46_F_12_N_5_O_2_OsP_3_: C, 45.04 (45.10); H, 3.86 (4.00); N, 5.84 (5.76). UV–vis in 50% aqueous CH_3_CN, l_max_ [nm] (e[mol^−1^dm^3^cm^−1^]): 269 (33580), 330 (12680), 376 (16150), 524 (6650), 670 (7530).

### 4.3 X‐ray Crystallography

Single crystals of **2**–**6** were sealed in a loop and mounted on a goniometer head. Data collection was performed at 100 K using a Bruker SMART APEX II CCD area detector system with a graphite‐monochromated Mo Kα radiation (*λ* = 0.71073 Å) in the *ω*‐scan mode. Data acquisition and reduction were performed using the Bruker APEX3 software suite. The structures were solved with SHELXT‐2015 and refined using SHELXL‐2014 or ‐2017 within the Olex2 program package [57, 58, 59].

CCDC 2484000–2484004 contains the supplementary crystallographic data for this paper. These data can be obtained free of charge from The Cambridge Crystallographic Data Centre via www.ccdc.cam.ac.uk/data_request/cif.

### 4.4 EPR Study

DMPO (5,5‐dimethyl‐1‐pyrroline N‐oxide) was employed as a spin‐trap agent for •OH in vitro. Various sample solutions containing (i) 200 µM of 5 and 100 mM H_2_O_2_; (ii) 200 µM of 5; and (iii) 100 mM H_2_O_2_ were mixed with 100 mM DMPO in CH_3_CN. The solution was dispersed for 6 h, transferred into a quartz capillary rapidly and detected by a Bruker EMX‐plus at room temperature.

### 4.5 Cell Lines and Cell Culture Conditions

The cell lines used in this study included human lung cancer (NCI‐H460), human lung carcinoma (A549), hepatocellular carcinoma (HepG2), human colon cancer (HCT116), mouse colon carcinoma (CT26) and mouse breast adenocarcinoma (4T1). All cell lines were cultured at 37 °C in a humidified atmosphere containing 5% CO_2_. NCI‐H460, A549, HepG2, HCT116, CT26 and 4T1 cells were cultured in Roswell Park Memorial Institute medium (RPMI 1640; Gibco), supplemented with 1% penicillin‐streptomycin (Gibco), 1% GlutaMAX (Gibco), and 10% fetal bovine serum (Gibco). Normal human lung fibroblast cells (MRC5) were cultured in Minimum Essential Medium (MEM) containing 10% fetal bovine serum, 1% penicillin‐streptomycin, and 1% GlutaMAX.

NCI‐H460 (SCSP‐584), A549 (SCSP‐503), HepG2 (SCSP‐510) and HCT116 (SCSP‐644) were purchased from the Cell Bank of Type Culture Collection, Chinese Academy of Sciences, Shanghai, China. 4T1 (CL‐0007) and CT26 (CL‐0071) were purchased from Procell, Wuhan, China. MRC5 (CX0053) cell line was purchased from Boster Biological Technology, Wuhan, China. All cell lines were authenticated by STR profiling and were regularly tested to exclude mycoplasma contamination.

### 4.6 Antiproliferative Activity Assessment

The tested compounds were freshly dissolved in DMSO to prepare stock solutions, while cisplatin was dissolved in 0.9% NaCl. Antiproliferative activity was determined using the naphthol blue black (NBB) assay. Cells were seeded in 96‐well plates and cultured for 12–24 h. The cells were then treated with varying concentrations of the compounds (final DMSO concentration <1%) for 48 h. Following incubation, the media was removed, and the cells were fixed with 50 µl of 3% formaldehyde in PBS for 15 min. Cells were then stained with a solution containing 0.05% naphthol blue black (Sigma) in 9% acetic acid with 0.1 M sodium acetate (50 µl/well) for overnight. After carefully removing the staining solution, the cells were gently washed with deionized water and solubilized in 50 mM NaOH solution (100 µl/well). Absorbance was measured at 590 nm using a microplate reader (Infinite 200PRO M Nano, TECAN, Switzerland). Antiproliferative activity was expressed as the half inhibitory concentration (IC_50_) relative to untreated controls. IC_50_ values were calculated from concentration‐effect curves by logarithmic interpolation using Origin. Every IC_50_ value represents the means of three independent experiments, with three replicates per concentration.

For inhibitor studies, cells were pre‐treated for 2 h with specific inhibitors: Z‐VAD‐FMK (20 µM), Ferrostatin‐1(10 µM). Cells were then exposed to various concentrations of the test compounds for another 46 h, followed by the NBB assay as described above. The effect of each inhibitor on cell viability was subsequently evaluated.

### 4.7 Analysis of GSH, ROS, and LPO

#### 4.7.1 GSH Measurement

Intracellular GSH levels were quantified using a commercial glutathione assay kit. Briefly, NCI‐H460 cells were treated with varying concentrations of osmium complex. Post‐treatment, cells were subjected to repeated freeze‐thaw cycles (at least three times) to ensure complete lysis. The lysates were then centrifuged, and the resulting supernatants were collected for GSH analysis. Absorbance was measured at 412 nm, and the concentration of reduced GSH in each sample was calculated using a standard curve and normalized to the total protein content.

#### 4.7.2 ROS Detection

Intracellular ROS levels were determined using a ROS assay kit. NCI‐H460 cells were treated with different concentrations of the osmium complex. Post‐treatment, the culture medium was removed and the cells were washed with PBS, followed by incubation with 10 µM DCFH‐DA in FBS‐free medium for 20 min at 37 °C. After staining, the cells were washed three times with FBS‐free medium, collected and analyzed by flow cytometer.

#### 4.7.3 LPO Quantification

Intracellular LPO levels were measured using a LPO assay kit. NCI‐H460 cells were exposed to varying concentrations of the osmium complex. Post‐treatment, cells were treated in accordance with the standard protocol for the LPO assay kit. LPO concentrations were determined using a standard curve and normalized to total protein content.

### 4.8 Western Blot Analysis

The following primary antibodies were used: GAPDH (CST, #2118), *β*‐Actin (CST, #4970), Cleaved PARP1 (Abcam, ab4830), Caspase‐8 (CST, #4790), Cleaved‐Caspase‐3 (Abcam, ab32042), BiP (CST, C50B12), CHOP (CST, #5554), Phospho‐eIF2a(Ser51) (CST, #3398), GPX4 (CST, #52455), FSP1 (SANTA‐CRUZ, sc‐377120). Protein expression levels in NCI‐H460 cells after treatment with the compounds were assessed by western blot assay. Briefly, cells were seeded and allowed to adhere for 12–24 h, then treated with varying concentrations of osmium complex for 24 h. After treatment, cells were lysed and protein concentrations were determined using a BCA protein assay kit (measured at 562 nm). Equal amounts of cellular proteins were denaturated and separated on 10%–15% separation gels, followed by transfer onto nitrocellulose membranes. Membranes were blocked for 40 min at room temperature and then incubated overnight at 4 °C with the appropriate primary antibodies. After washing, membranes were incubated with the secondary antibodies for 1.5 h at room temperature. Protein bands were visualized using an ECL Plus detection kit and imaged on a High ChemiDoc XRS (Bio‐Rad ChemiDoc XRS+, USA).

### 4.9 Dead/Live Cell Co‐Staining

The therapeutic efficiency of treatments was evaluated using dead/live cell co‐staining. Briefly, NCI‐H460 cells were seeded onto confocal plates and incubated for 24 h at 37°C. Cells were then treated with medium containing osmium compounds and incubated for an additional 24 h. After treatment, cells were stained with a dye solution containing Calcein AM and Propidium Iodide (PI) for 30 min at 37°C. Imaging was carried out using an inverted fluorescence microscope.

### 4.10 Flow Cytometric Analysis of Apoptosis Evaluation

Apoptosis was assessed using a FITC Annexin V Apoptosis Detection Kit. Briefly, NCI‐H460 cells were seeded in six‐well plates and allowed to adhere for 24 h at 37°C. Cells were then incubated with the medium containing osmium compounds for an additional 24 h. After treatment, the cells were collected, centrifuged at 1000 rpm for 5 min, and washed twice with PBS. The cell pellet was resuspended in 300 µl 1X annexin‐binding buffer and stained with 5 µl annexin V and 5 µl propidium iodide for 10 min at room temperature. Prior to analysis, 200 µl 1X annexin‐binding buffer was added, and the samples were filtered. Samples were stored at 4°C protected from light and analysed by a flow cytometer within 1 h.

### 4.11 DAMPs of Immunogenic Cell Death

#### 4.11.1 Flow Cytometric Analysis of Cell Surface CRT

Cell surface exposure of CRT was evaluated by flow cytometry. Briefly, NCI‐H460 cells were treated with varying concentrations of osmium complex for 24 h. Post incubation, cells were collected, washed twice with PBS, and incubated with a primary anti‐CRT antibody (CST) diluted in cold blocking buffer (3% BSA in PBS) for 2 h on ice. After washing, cells were incubated with goat anti‐rabbit IgG Alexa Fluor 488 secondary antibody (Invitrogen A‐11008) in blocking buffer for 1 h at room temperature in the dark. Cells were washed with PBS and stained with PI in PBS for 10 min at room temperature. Samples were immediately analysed by flow cytometry.

#### 4.11.2 Detection of HMGB1 Release by ELISA Assay

NCI‐H460 cells were seeded at a density of 2.5 × 10^5^ cells/ml in 12‐well plates and incubated until confluent (approximately 24 h), until approximately 80% confluent. Cells were then incubated with respective treatments for 24 h. Following treatment, supernatants were collected from each well (3 wells/group) and centrifuged to remove cell debris. HMGBl levels in the supernatant were determined using the HMGB1 Detection ELISA Kit (Chondrex, #6010) according to the manufacturer's instructions. Absorbance was measured using an Infinite200PRO M Nano plate reader.

#### 4.11.3 Extracellular ATP Content Detection

NCI‐H460 cells were exposed with varying concentrations of the osmium complex for 24 h. After incubation, the culture supernatants were transferred to white‐walled, opaque 96‐well plates. An equal volume of ATP assay reagent (Cell Titer Glo Luminescent Cell Viability Assay‐ATP, Promega) was added to each well, and the plate was mixed thoroughly according to the manufacturer's instructions. Luminescence was measured using a GloMax Navigator Microplate luminometer to quantify the intracellular ATP levels.

#### 4.11.4 Immunofluorescent Detection of CRT

For cell surface CRT detection, tumor cells were harvested and seeded at a density of 3 × 10^4^cells/chamber overnight in Cellvis Chamber Slide‐8 Chamber. Cells were then incubated with the respective treatments at 37 °C for 12 h. Post incubation, the media was removed, and cells were washed (3x) with PBS. Cells were then fixed with 4% paraformaldehyde for 20 min at RT. For staining, cells were incubated with a primary CRT antibody (CST, #12238) in blocking buffer (3%BSA/PBS) at a 1:400 dilution for 3 h on ice. Following three washes with cold PBS, cells were incubated with an appropriate Alexa 488‐conjugated secondary antibody diluted in a cold blocking buffer for 30 min. Subsequently, cells were washed (3x) with PBS stained with 1,1'‐dioctadecyl‐3,3,3',3'‐tetramethylindocarbocyanine perchlorate (Dil) and Hoechst 33342 in PBS for 20 min at RT. Finally, cells were directly imaged fluorescently on a Zeiss LSM900 laser confocal microscope.

#### 4.11.5 Immunofluorescent Detection of HMGB1 Levels In Cells

For intracellular *HMGB1* staining, cells were washed with PBS, fixed with 4% paraformaldehyde for 20 min, and permeabilized with 0.1% Triton X‐100 (Sigma) for 10 min. After rinsing three times with PBS, nonspecific binding sites were blocked with 10% fetal bovine serum in PBS for 30 min. Cells were then incubated with the primary antibody for l h, followed by three washes with PBS and incubation with an Alexa Fluor 488‐conjugated secondary antibody (1:500, Molecular Probes, Carlsbad, CA, USA) for 30 mins. Stained cells were mounted on slides and visualized by fluorescence microscopy.

### 4.12 Tissue Distribution of Osmium by ICP‐MS

To evaluate the biodistribution of osmium complexes, tumor‐bearing mice from CT26 and NCI‐H460 xenograft models were employed. In the CT26 model, BALB/c mice bearing subcutaneous CT26 tumors received intravenous injections of Os(IV) (5 mg/kg) every 3 days for a total of five doses. At the experimental endpoint (day 14), mice were euthanized, and major organs (heart, liver, spleen, lung, kidney) and tumors were harvested. For the NCI‐H460 model, BALB/c nude mice bearing subcutaneous NCI‐H460 tumors were intravenously administered with either Os(III) or Os(IV) (5 mg/kg) every 4 days for five doses, and tissues were collected on day 20. All tissue samples were weighed and digested with aqua regia (HCl:HNO_3_ = 3:1, v/v) at 80 °C for 24 h. The resulting solutions were diluted with ultrapure water and analyzed by inductively coupled plasma mass spectrometry (ICP‐MS, Agilent 7900) to quantify osmium content. Osmium concentrations were expressed as micrograms per gram of tissue (µg/g).

### 4.13 In Vitro Hemocompatibility

The in vitro hemocompatibility of **Os** compounds was evaluated by incubating with red blood cells (RBCs). Briefly, RBCs were centrifugation and washed three times with PBS. The RBCs obtained in this way were dispersed in an appropriate volume of PBS. Various concentrations of **Os** compounds were mixed with an equal volume of RBCs in a centrifuge tube and incubated at 37°C for 24 and 48 h. The mixture was centrifuged at 1000 × g for 10 min, and then 30 µL of the supernatant was diluted with 100 µL of PBS in a 96‐well plate. PBS and 1% Triton X‐100 treatments served as negative and positive controls, respectively. OD value was measured at 540 nm using a microplate reader.

Calculation of the hemolysis rate:

$$\begin{array}{ccc} & & \mathrm{Hemolysis}\left(\%\right)\\ & & \;=\left[\left(OD_{\mathrm{Samp}\mathrm{le}}-OD_{\mathrm{PBS}}\right)/\left(OD_{\mathrm{Trit}\mathrm{on}\;X-100}-OD_{\mathrm{PBS}}\right)\right]\times 100\% \end{array}$$

### 4.14 In Vivo Therapeutic Evaluation

#### 4.14.1 Laboratory Animal and Ethics Statement

All animal experiments were approved by the Institutional Animal Care and Use Committee of Shantou University Medical College and conducted in accordance with the institutional guidelines (Certificate NO. SYXK2022‐0079). BALB/c mice used in this study were obtained from Guangdong Vital River Laboratory Animal Technology Co. Ltd (Guangdong, China).

#### 4.14.2 Tumor Growth Assessment

Tumor volume (V) was calculated using the formula:

V = W^2^ × L/2, where W and L are the width and length of the tumor, respectively.

#### 4.14.3 Tumor Growth Inhibition Rate

Tumor growth inhibition rate was determined using the following formula:

Tumor growth inhibition rate (%) = (1‐mean tumor weight of the treatment/mean tumor weight of the negative group) × 100%. All data were expressed as mean ± SD.

Statistical analyses were performed using SPSS software

#### 4.14.4 NCI‐H460 Xenograft Nude Mouse Model

To establish a xenograft tumor model, female BALB/c‐nude mice (5 weeks old) were subcutaneously injected with 3.5 × 10^6^ NCI‐H460 cells suspended in PBS. When tumors reached approximately 100 mm^3^, mice were randomized into five groups (n = 4 per group): Control (10%PET), Oxaliplatin (3 mg/kg), complex 5 (5 mg/kg), and complex 6 (5 mg/kg). Treatments were administered via tail vein injection on days 0, 4, 8, 12, and 16. Tumor volume and body weight of mice were measured every two days. On day 20, at the end of the treatment period, mice were sacrificed, and tumors were excised and weighed. Major organs and tumor tissues were collected for H&E staining and subsequent histological analysis.

#### 4.14.5 In Vivo Vaccination and Rechallenge Experiment

4T1 cells were seeded and incubated with or without the indicated compounds for 24 h. After incubation, all cells were collected. Female BALB/c mice were randomly divided into four groups (n = 5 per group) and immunized subcutaneously in the left flank on day −10 with 2 × 10^6^ 4T1 cells that had undergone the respective treatments. More specifically, mice (*n*  = 5 per group) were inoculated subcutaneously with 4T1 cells (2 × 10^6^) pretreated for 24 h with: freeze‐thawed necrotic 4T1 cells (negative control, Group I), doxorubicin (30 µM, ICD‐positive control, Group II), **Os(III)** (10 µM, Group III), and **Os(IV)** (10 µM, Group IV). On day 0, all mice were rechallenged by subcutaneous injection of 7.5 × 10^5^ 4T1 cells into the right flank. Rechallenged tumor volumes were measured every two days.

#### 4.14.6 CT26 Homologous Transplant Mouse Model

To establish a CT26 xenograft tumor model, female BALB/c mice (5 weeks old) were subcutaneously injected with 1 × 10^6^ CT26 cells suspended in PBS. When tumor volumes reached approximately 100 mm^3^, mice were randomized into different groups (n = 4 per group): Control (10% PET), Oxaliplatin (3 mg/kg, 5% Glucose), **Os(IV)** (5 mg/kg, 10% PET). Treatments were administered via tail vein injection on days 0, 3, 6, 9, and 12. Tumor volume and body weight were measured every two days. At the end of treatment (day 15), mice were scarified and tumors were excised, measured, and weighed. Major organs were collected for H&E staining, and tumors were collected for further immunoassays.

### 4.15 Histological Analysis

Collected organs and tumor tissues were fixed with 10% paraformaldehyde for 24 h at room temperature, dehydrated overnight in an automated tissue processor, and embedded in paraffin. Paraffin‐embedded tissues were cut into 4 µm‐thick slices for H&E staining or immunohistochemical analysis.

#### 4.15.1 H&E Staining

Sections were baked for 1–2 h, deparaffinization in xylene, and rehydrated through a graded ethanol series (100%, 95%, 90%, and 80%). Following hydration, sections were stained with H&E, dehydrated through increasing ethanol concentrations (80%, 95%, and 100% ethanol), cleared in xylene, and mounted with a resinous medium. Stained slides were examined using a digital pathology slide scanner (KF‐PRO‐005, KFBIO).

#### 4.15.2 Immunohistochemical Analysis

Sections were baked as described above, then rehydrated through graded ethanol. Endogenous peroxidase activity was blocked using 3% hydrogen peroxide for 10 min. Following antigen retrieval, slides were incubated with primary antibodies against mouse CD4 (MXB, product number: RMA‐0620, clone number: SP35, species: rabbit monoclonal antibody), CD8 (MXB, product number: RMA‐0514, clone number: SP16, species: rabbit monoclonal), Foxp3 (MXB, product number: RMA‐1004, clone number: 236A/E7, species: mouse monoclonal) or CD20 (MXB, product number: Kit‐0001, clone number: L26, species: mouse monoclonal) followed by detection using a secondary antibody conjugated to an enzyme. Visualization was achieved using a chromogen, with optional hematoxylin counterstaining. Finally, slides were dehydrated, cleared, and cover slipped for microscopic analysis to assess antigen presence and distribution.

### 4.16 In Vivo Anti‐Tumor Immunity Analysis by Flow Cytometry

After the end of treatment, all mice were euthanized. Tumors, lymph nodes, and spleens were harvested, mechanically dissociated in PBS, and single‐cell suspensions were prepared by filtering through a 70 µm cell strainer. The cell suspensions were centrifuged at 200 g for 5 min, and the supernatant was discarded. Cells were resuspended in staining buffer and gently mixed. For live/dead discrimination, 1 µL of Fixable Viability Stain 700 was added to each sample and mixed thoroughly. To block nonspecific antibody binding, 2 µL of FC blocking (1 µg/10^6^ cells) was added, and samples were mixed and incubated on ice for 20 min. After incubation, cells were washed by centrifugation (200 g, 5 min) and resuspended in 100 µL staining buffer for antibody labelling. For T cell analysis, cells were stained with BV786 Rat Anti‐Mouse CD45, BV510‐anti‐mouse CD3, APC‐anti‐mouse CD4, and PE‐anti‐mouse CD8 antibodies. For dendritic cell analysis (CD11C^+^CD80^+^CD86^+^ phenotype), cells were stained with APC‐Cy7 Hamster Anti‐Mouse CD11c, PerCP‐Cy5.5 Rat Anti‐Mouse I‐A/I‐E, FITC Anti‐Mouse CD80, and FITC Anti‐Mouse CD86. Flow cytometry data were acquired on a CYTEK Aurora flow cytometer and analysed using FlowJo software.

### 4.17 Statistical Analysis

Gray values for Western blots were quantified using Bio‐Rad Image Lab software and Fluorescence intensities were quantified using the intensity measurement tool in Zeiss ZEN software. Data were presented as mean ± SD and analyzed by *t‐*test.

## Conflicts of Interest

The authors declare no conflict of interest.

## Supporting information




**Supporting File 1**: advs75576‐sup‐0001‐SuppMat.pdf.

**Supporting File 2**: advs75576‐sup‐0002‐Data.zip.

## Data Availability

The data that support the findings of this study are available from the corresponding author upon reasonable request.
