# Supplementary material for: A Catalytic Osmium Redox Couple Collapses Cancer Redox Balance
Source: Adv Sci (Weinh). 2026 May 8;13(43):e75576. doi: 10.1002/advs.75576 (PMC13336027; doi:10.1002/advs.75576)
Supplement: Supplementary file 1 — Supporting File 1: advs75576‐sup‐0001‐SuppMat.pdf. [file ADVS-13-e75576-s002.pdf]

WILEY-VCH

Supporting information

A catalytic osmium redox couple collapses cancer redox balance

Wan-Qiong Huang, Tao Huang, Yiming Hao, Gui-Feng Huang, Yi-Lang Yan, Xin Fang, Ying Chen, Xiao-Long Wei, Wai-Lun Man,* and Wen-Xiu Ni**

Contents of the Supporting Information

Experimental Section **1**

Materials 1

Instrumentation 1

Solution Stability Analysis by UV–vis 2

Cellular uptake 2

Analysis of Mitochondrial Membrane Potential (MMP) 2

Analysis of Calcium ion 2

Results **3**

Table S1. IR (KBr) and CV (CH_3CN) data for 2–6. 3

Table S2. Crystal data and structure refinement details for 2–6. 3

Table S3. Selected bond distances of 2–6. 4

Table S4. IC_{50} values (48 h) of different complexes in various cell lines by means of NBB assay. 4

Figure S1. IR (KBr) of 2–6. Insets show the N–H stretches 5

Figure S2. ESI mass spectra of 2–6 in CH_3CN. 6

Figure S3. Hi-Res mass spectra of 2–6 in CH_2Cl_2. Observed (blue), calculated (black). 7

Figure S4. ORTEP diagram of 6 at a 50% probability level. 8

Figure S5. Stability of osmium complexes (25 μM) in DMSO. 8

Figure S6. Stability of 25 μM 2-6 in a medium (1% DMSO). 9

Figure S7. Cytotoxicity evaluation of Os^{III} series compounds. 9

Figure S8. Mechanistic insights analysis in 10

Figure S9. Western blot analysis of endoplasmic reticulum stress and apoptosis. 11

Figure S10. Flow cytometry results of apoptosis. 11

Figure S11. Oxidation of Os(III). 12

Figure S12. ROS detection in NCI-H460 cells. 12

Figure S13. GSH contents in NCI-H460 and MRC5. 13

Figure S14. Os(III) or Os(IV) efficiently induce the death of cancer cells. 13

Figure S15. Induction of apoptosis of Os(III) or Os(IV). 14

Figure S16. Gray value quantification of apoptosis-related proteins in Figure 4e. 14

Figure S17. Immunofluorescence of GPX4 protein. 15

Figure S18. Immunofluorescence of FSP1 protein. 15

Figure S19. Representative western blot analyses of GPX4 and FSP1. 16

Figure S20. Cell viability in 24 h under incubation of Os(III) and inhibitors. 16

Figure S21. Cell viability in 24 h under incubation of Os(IV) and inhibitors. 17

Figure S22. Gray value quantification of ER stress-related proteins in Figure 4h 17

Figure S23. Flow cytometry results of a Ca^{2+} assay. 18

Figure S24. Confocal images of intracellular Ca^{2+} assays with Fluo-4 AM. 18

Figure S25. CRT translocation analysis. 19

Figure S26. HMGB1 secretion analysis. 19

Figure S27. Release of HMGB1 in cell culture supernatant. 20

Figure S28. Antitumor effect of Os compounds on the NCI-H460 model. 21

Figure S29. Hematoxylin−Eosin staining of tumors in different treatments on NCI-H460 model. 22

Figure S30. Hematoxylin−Eosin staining of major organs in different treatments on NCI-H460 model. 23

Figure S31. Hematoxylin−Eosin staining of major organs in different treatments on CT26 model. 24

Figure S32. Immune response analysis of Os(IV) on CT26 model ($CD20^+$ B cells and Foxp3+ T cells) 24

Figure S33. Immune response analysis of Os(IV) on CT26 model ($CD4^+$ and $CD8^+$ T cells in spleen) 25

Figure S34. Immune response analysis of Os(IV) on CT26 model (DC cells in spleen). 25

Figure S35. The concentration of Os was quantified in major organs (heart, liver, spleen, lung, kidney) and tumors 25

Figure S36. Hemolysis of red blood cells seen in the presence of various concentrations of Os(III) (a) and Os(IV) (b) 26

Raw data of Western blot 27

Figure S37. Unprocessed original membranes of Western blot shown in Figures 4e and 4h. 30

Figure S38. Unprocessed original membranes of Western blot shown in Figure S9. 31

Figure S39. Unprocessed original membranes of Western blot shown in Figure S19. 32

Experimental Section

Materials

Chemical was used as received unless specified otherwise. Naphthol blue black (NBB) and Cisplatin were purchased from Alfa. GSH was purchased from Arcros. NAD^+ was purchased from sigma. FITC Annexin V Apoptosis Detection Kit were obtained from BD Pharmingen™. Reactive Oxygen Species Assay Kit, Mitochondrial membrane potential assay kit with JC-1, Fluo-4 Calcium Assay Kit, Calcein/PI Assay Kit were purchased from Beyotime. Inhibitor Z-VAD-FMK and Ferrostatin-1 were purchased from MCE. 5,5-dimethyl-1-pyrroline N-oxide (DMPO) was bought from Macklin. Lipid hydroperoxide (LPO) Kit and Glutathion reductases assay kit were purchased from Beijing Solarbio Science & Technology Co., Ltd.

Instrumentation

UV-vis spectra were recorded on a Shimadzu UV2450-2550 or Agilent Cary 8454 UV-visible spectrophotometer. Infrared spectra were obtained from KBr plates using a Nicolet AVATAR 360 FTIR spectrophotometer. ESI-MS was conducted on an AB SCIEX 3200 Qtrap mass spectrometer. High-resolution mass spectrometry (HRMS) was conducted using Q Exactive Mass Spectrometers (Thermo Fisher). Single crystal data were collected at 100 K using a Bruker SMART APEX II CCD area detector system. Elemental analysis was done on a Vario EL cube CHNs analyzer (Elementar). EPR results were obtained from an electron paramagnetic resonance spectrometer (EMXplus, Bruker). Intracellular osmium accumulation was measured by ICP-MS (Agilent 7900). The absorption was recorded with a microplate reader (Infinite 200PRO M Nano, TECAN). Analytical flow cytometry was used by BD Accuri™ C6 or CYTEK Aurora™. Western blot was visualized with an imaging system (Bio-Rad ChemiDoc XRS+ or Tanon-5200CE). The fluorescence images were recorded with ZEISS LSM900 or Axio Observer A1. Luminescence was measured using a GloMax Navigator Microplate luminometer (PROMEGA). Histological images were visualized with a digital pathology slide scanner (KF-PRO-005, KFBIO).

Solution Stability Analysis by UV–vis

The stability of all osmium compounds in DMSO was examined using UV-Vis spectroscopy. A 10 mM stock solution in DMSO was prepared and diluted to a working concentration of 25 μM and analyzed with a UV-Vis spectrometer. The osmium compounds (25 μM) were also prepared in 1% DMSO/Medium. The changes in the spectrum of the corresponding solution were recorded at different time intervals by a UV-vis absorption spectrometer.

Cellular uptake

NCI-H460 cells in the logarithmic growth phase were collected. The cells were seeded in six-well plates at a density of 2.5×10^5 cells/mL and cultured at 37°C/5% CO_2 for 24 h. After treatment with different concentrations (three replicates) of the tested compounds for 6 h, cells were washed twice (PBS) and collected. Cells were lysed and protein concentrations were measured using the BCA Protein Assay Kit at 562 nm by Infinite M200. The samples were digested in 65% HNO_3 at room temperature for 24 h. Each sample was diluted with Milli-Q H_2O to obtain 3% HNO_3 solutions. The standards for calibration were freshly prepared by diluting an Os standard solution (1000 ppm) with Milli-Q H_2O. The osmium concentration in each sample was determined by ICP-MS.

Analysis of Mitochondrial Membrane Potential (MMP)

Mitochondrial Membrane Potential (MMP) was determined using a JC-1 Assay kit. In brief, NCI-H460 cells were exposed to varying concentrations of the osmium complex. Post-treatment, the media was removed, and cells were washed with PBS. The cells were collected and resuspended in 1 mL JC-1 dyestuff and placed into a cell incubator for 20 min. Subsequently, the cells were rinsed twice with pre-cooling JC-1 staining buffer and measured by flow cytometry.

Analysis of Calcium ion

Intracellular Calcium ion concentration was determined using a Fluo-4 Calcium Assay Kit. In brief, NCI-H460 cells were exposed to varying concentrations of the osmium complex. Post-treatment, each plate was incubated with a Fluo-4 Staining Solution prepared by the standard protocol. After co-staining for 30 min at 37 °C, imaging was carried out via fluorescence microscopy. For flow cytometry analysis, the cells were collected and washed with PBS, and then the cell samples were incubated with a Fluo-4 Staining Solution prepared by the standard

protocol. After co-staining for 30 min at 37 °C, the fluorescence was measured by flow cytometry.

Table S1. IR (KBr) and CV (CH_3CN) data for **2–6.**

Complex	KBr (cm^{-1}) v(N–H)	$E_{1/2}$ (volts vs $Fe^{+/0}$) $Os^{IV/III}$	$Os^{III/II}$
**2**	3338	–0.07	–1.34
**3**	3343	0.00	–1.06
**4**	3345	–0.08	–1.37
**5**	3338	–0.17	–1.46
**6**	3336	–0.17	–1.46

Table S2. Crystal data and structure refinement details for **2–6.**

	**2**	**3**	**4**	**5**	**6**
Empirical formula	$C_{43}H_{41}F_6N_4O_2OsP_2$	$C_{46}H_{43}F_6N_6O_2OsP_2$	$C_{47}H_{49}F_6N_4O_3OsP_2$	$C_{45}H_{46}F_6N_5O_2OsP_2$	$C_{47}H_{49}F_{12}N_6O_2OsP_3$
Formula weight	1011.94	1078.00	1084.04	1055.01	1241.03
Temperature/K	100.00	100.00	100.00	100.00	100.00
Crystal system	triclinic	triclinic	triclinic	monoclinic	triclinic
Space group	P-1	P-1	P-1	$P2_1/c$	P-1
a/Å	10.6379(9)	11.1414(14)	13.2768(12)	11.3834(8)	10.4468(14)
b/Å	14.0409(13)	15.4645(17)	13.6526(12)	20.7515(16)	14.160(2)
c/Å	14.2793(12)	15.7631(19)	13.6724(13)	18.7290(15)	16.395(3)
α/°	80.334(3)	111.659(4)	76.554(3)	90	97.070(6)
β/°	82.772(3)	103.229(5)	67.728(3)	105.793(3)	93.603(5)
γ/°	77.984(3)	105.113(4)	79.952(3)	90	93.446(5)
Volume/$Å^3$	2047.3(3)	2271.3(5)	2220.3(4)	4257.2(6)	2396.5(6)
Z	2	2	2	4	2
$\rho_{calc}mg/mm^3$	1.642	1.576	1.622	1.646	1.720
F(000)	1006.0	1074.0	1086.0	2108.0	1236.0
Reflections collected	117605	143367	108363	90808	105973
Independent reflections	10111 [R_{int} = 0.0382, R_{sigma} = 0.0212	11260 [R_{int} = 0.0556, R_{sigma} = 0.0227	11030 [R_{int} = 0.0801, R_{sigma} = 0.0384	10584 [R_{int} = 0.0641, R_{sigma} = 0.0342	11846 [R_{int} = 0.0526, R_{sigma} = 0.0276
Data/restraints/parameters	10111/0/523	11260/0/569	11030/0/571	10584/0/552	11846/0/646

Goodness-of-fit on F^2	1.093	1.096	1.041	1.038	1.044
Final R indexes [I>=2sinσ (I)]	R_1 = 0.0191, wR_2 = 0.0461	R_1 = 0.0368, wR_2 = 0.1138	R_1 = 0.0312, wR_2 = 0.0801	R_1 = 0.0245, wR_2 = 0.0513	R_1 = 0.0294, wR_2 = 0.0784
Final R indexes [all data]	R_1 = 0.0193, wR_2 = 0.0462	R_1 = 0.0381, wR_2 = 0.1146	R_1 = 0.0347, wR_2 = 0.0825	R_1 = 0.0293, wR_2 = 0.0529	R_1 = 0.0316, wR_2 = 0.0796

Table S3. Selected bond distances of **2–6.**

	**2**	**3**	**4**	**5**	**6**
Os1–N1	1.985(3) Å	1.993(4) Å	1.998(2) Å	1.991(2) Å	1.992(2) Å
Os1–N2	2.004(3) Å	1.991(4) Å	1.988(3) Å	1.987(2) Å	2.000(2) Å
Os1–N3	2.058(3) Å	2.053(4) Å	2.068(3) Å	2.069(2) Å	2.027(2) Å
Os1–N4	2.092(3) Å	2.078(4) Å	2.113(2) Å	2.103(2) Å	2.113(2) Å
Os1–O1	2.028(3) Å	2.035(3) Å	2.023(2) Å	2.039(2) Å	1.979(2) Å
Os1–O2	2.037(2) Å	2.019(3) Å	2.024(2) Å	2.018(2) Å	1.981(2) Å
P1–N3	1.610(3) Å	1.611(4) Å	1.609(3) Å	1.600(2) Å	1.635(2) Å

Table S4. IC_{50} values (48 h) of different complexes in various cell lines by means of NBB assay.

**Compound**	**2**	**3**	**4**	**5**	**6**	**Cisplatin**	**Oxaliplatin**
NCI-H460	2.3 ± 0.3	4.4 ± 0.4	1.6 ± 0.2	1.1 ± 0.1	0.5 ± 0.2	6.3 ± 1.0	1.8 ± 0.1
A549	2.3 ± 0.6	9.9 ± 1.1	1.3 ± 0.1	0.8 ± 0.4	0.6 ± 0.1	6.1 ± 0.2	8.7 ± 2.6
HCT116	1.6 ± 0.3	3.2 ± 1.2	0.7 ± 0.2	0.4 ± 0.2	0.3 ± 0.1	6.1 ± 0.5	3.0 ± 1.1
CT26	5.7 ± 0.4	12.6 ± 0.5	5.3 ± 0.8	3.9 ± 0.4	1.9 ± 0.2	6.7 ± 0.6	2.5 ± 0.2
HepG2	2.6 ± 0.5	6.3 ± 0.9	2.7 ± 0.5	1.5 ± 0.4	0.5 ± 0.1	5.8 ± 0.9	2.2 ± 0.5
4T1	2.0 ± 0.3	3.7 ± 0.3	0.8 ± 0.2	1.0 ± 0.3	0.6 ± 0.2	4.7 ± 0.1	5.8 ± 1.2
MRC5	6.7 ± 1.3	14.8 ± 1.5	4.8 ± 1.3	3.2 ± 0.7	4.6 ± 1.2	12.9 ± 4.2	>100

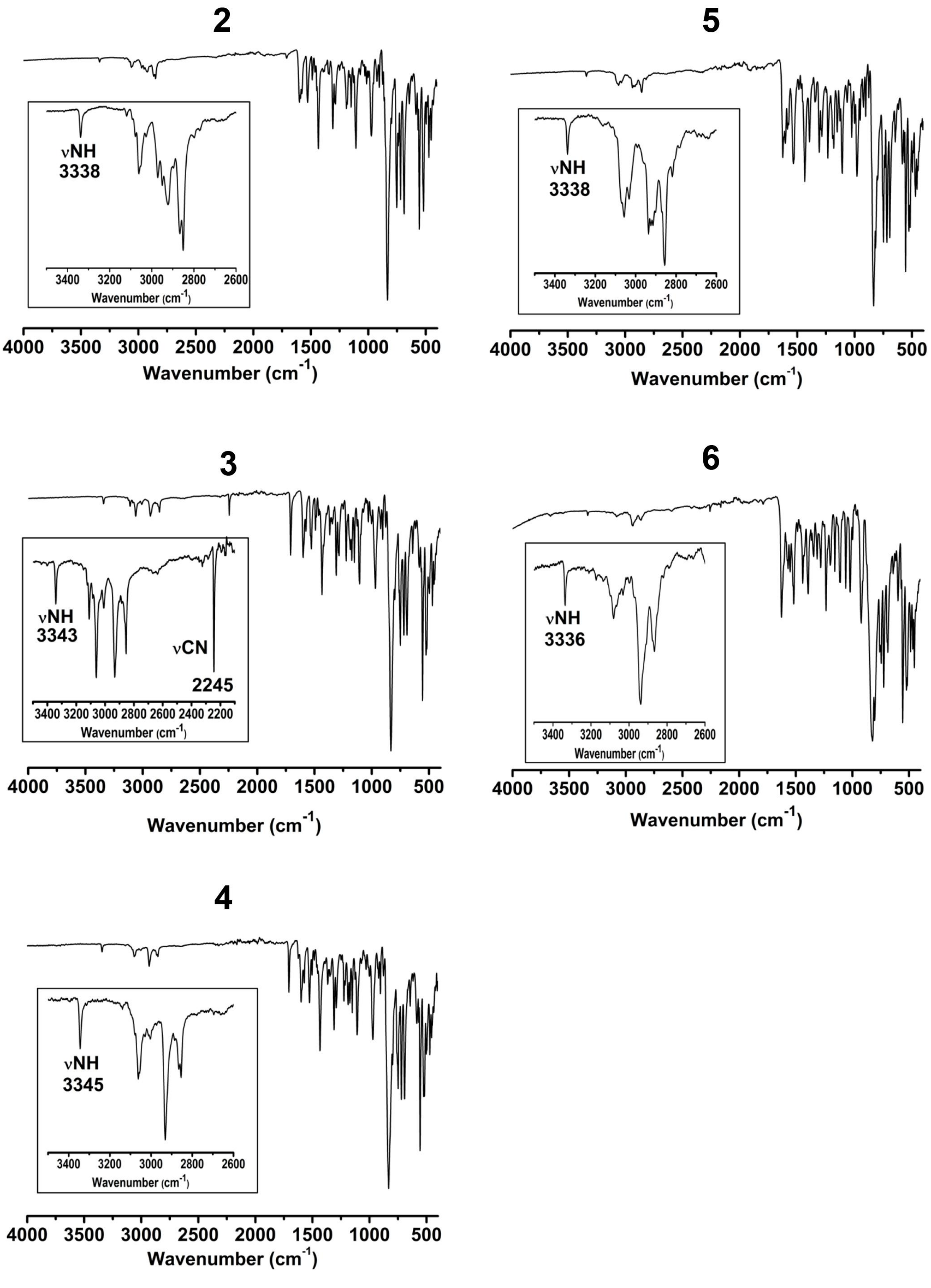

Figure S1. IR (KBr) of **2–6**. Insets show the N–H stretches

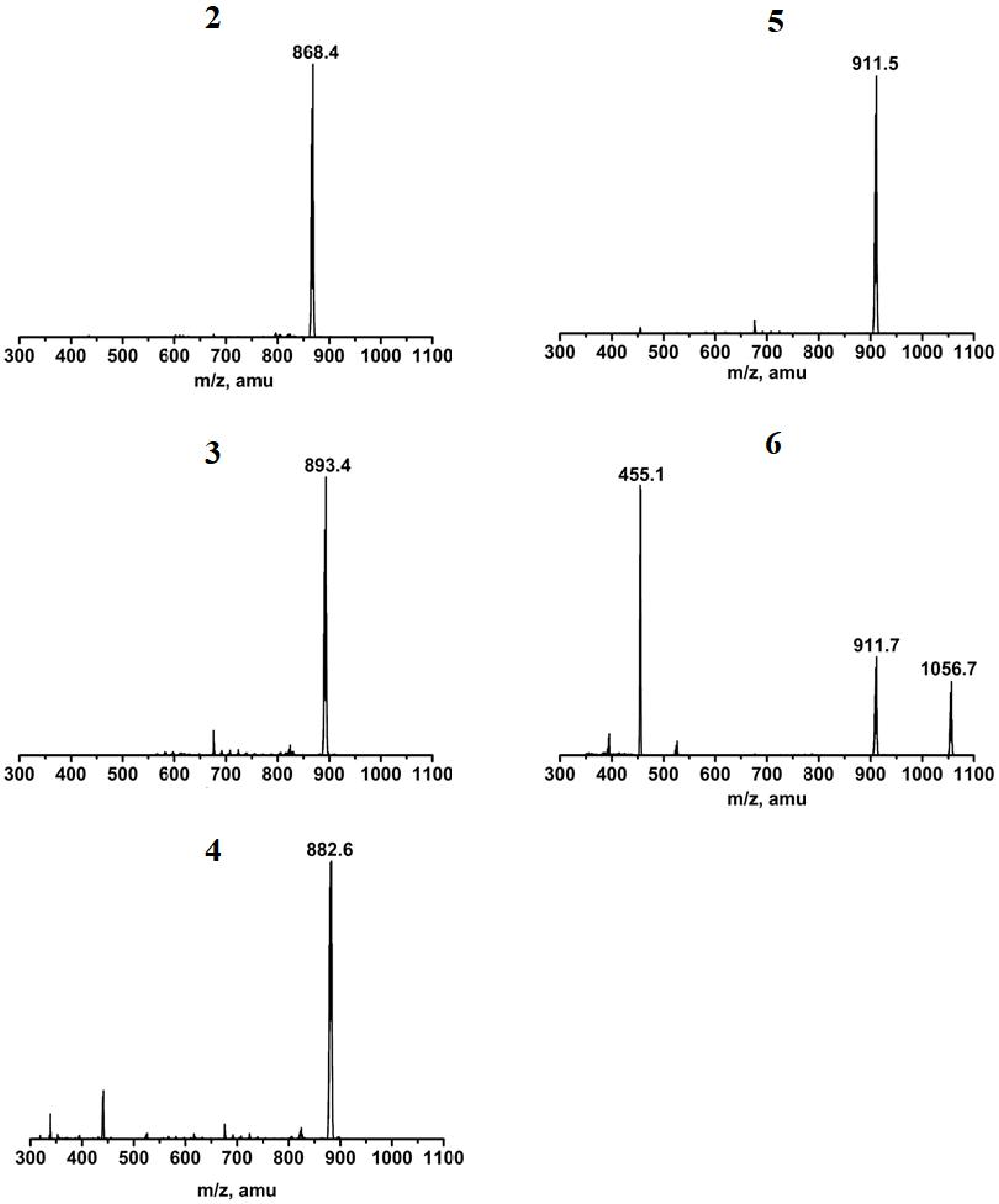

Figure S2. ESI mass spectra of **2–6** in CH_3CN.

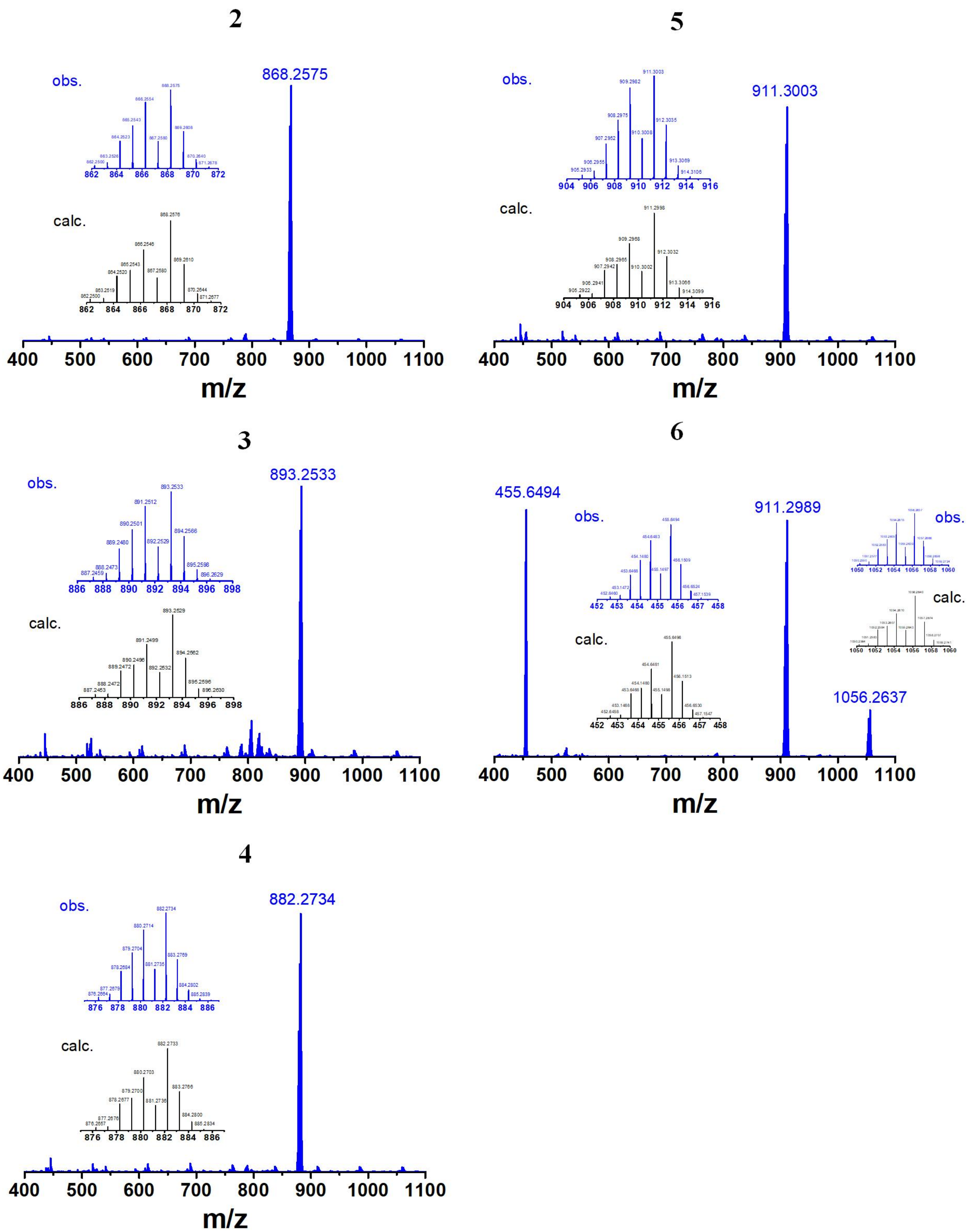

Figure S3. Hi-Res mass spectra of **2–6** in CH_2Cl_2. Observed (blue), calculated (black).

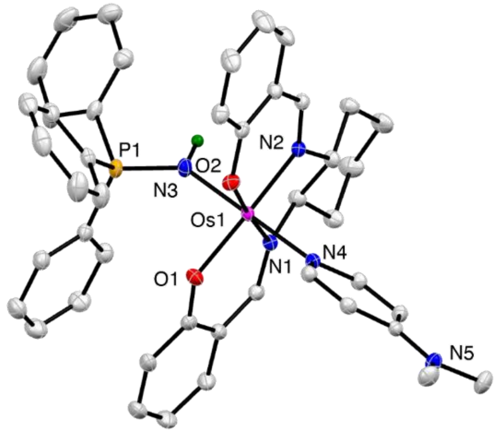

Figure S4. ORTEP diagram of **6** at a 50% probability level. H atoms (except N–H) are omitted for clarity.

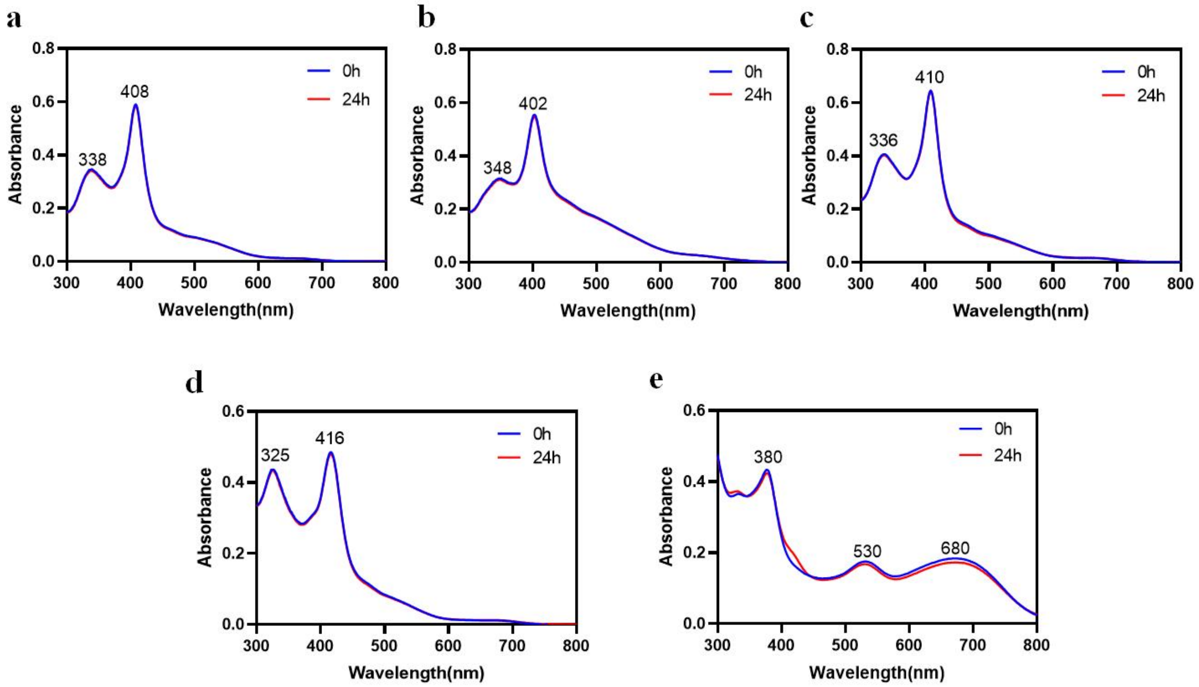

Figure S5. Stability of osmium complexes (25 μM) in DMSO. (a) **2**; (b) **3**; (c) **4**; (d) **5**; (e) **6**.

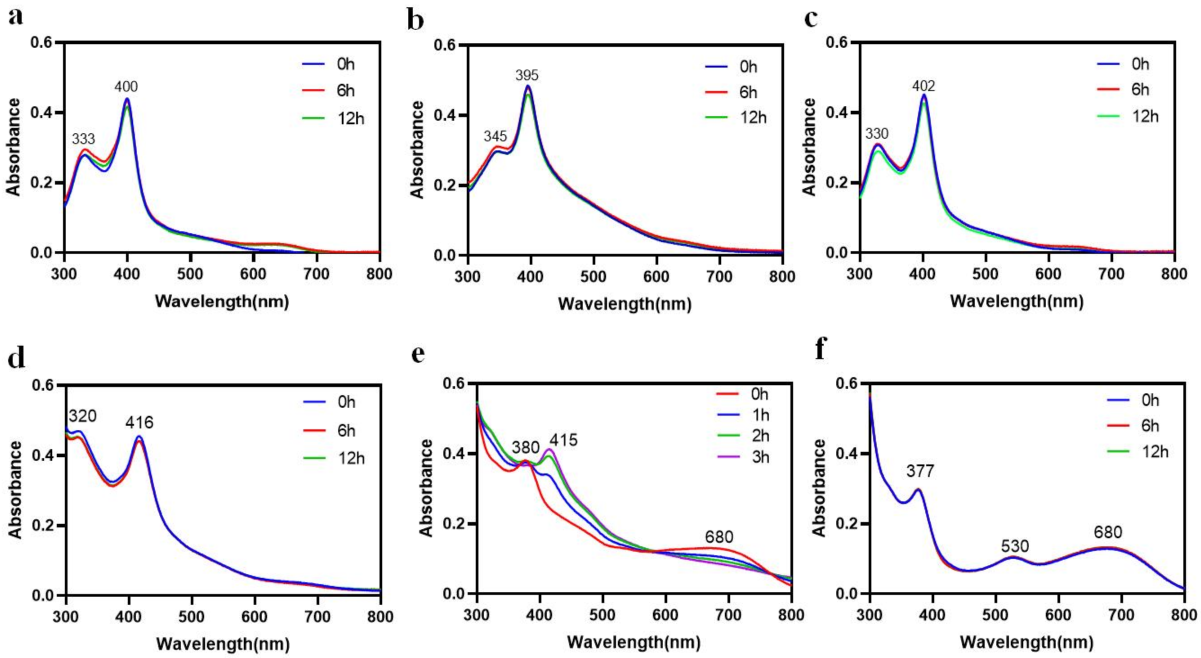

Figure S6. Stability of 25 μM **2-6** in a medium (1% DMSO). (a) **2**; (b) **3**; (c) **4**; (d) **5**; (e) **6**. (f) UV-Vis spectra of **6** (25 μM) in 10%PET-0.9%NaCl (PET: 60 % polyethylene glycol 400, 30 % ethanol, and 10 % Tween 80).

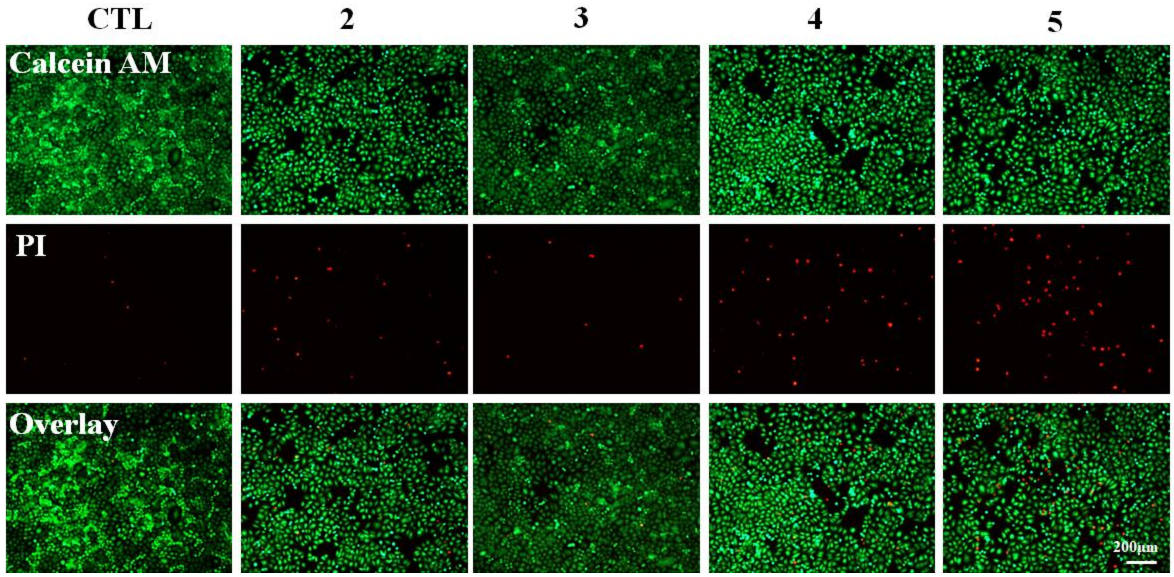

Figure S7. Cytotoxicity evaluation of Os^{III} series compounds. NCI-H460 cells treated with **2-5** at 4 μM for 24 h via live (green) and dead (red) staining. Scale bar: 200 μm.

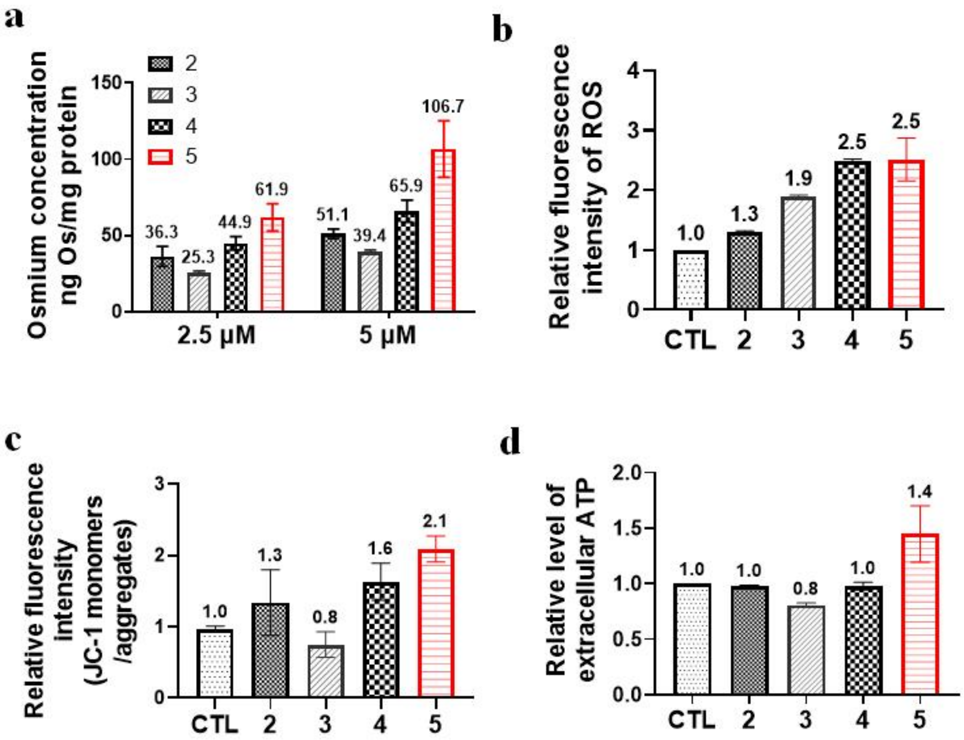

Figure S8. Mechanistic insights analysis in Os^{III}-treated NCI-H460 cells. Tested compounds were **2**, **3, 4,** and **5**. (a) ICP-MS detection of the osmium content. NCI-H460 cells were incubated with Os compounds (2.5 and 5 μM) for 6 h. (b) Flow cytometry results of a ROS generation assay. NCI-H460 cells treated with Os compounds (2 μM) for 6 h. (c) Flow cytometry results of a JC-1 assay. NCI-H460 cells were treated with Os compounds (4 μM) for 12 h. (d) Fluorescence intensity of extracellular ATP results. NCI-H460 cells were incubated with Os compounds (4 μM) for 24 h.

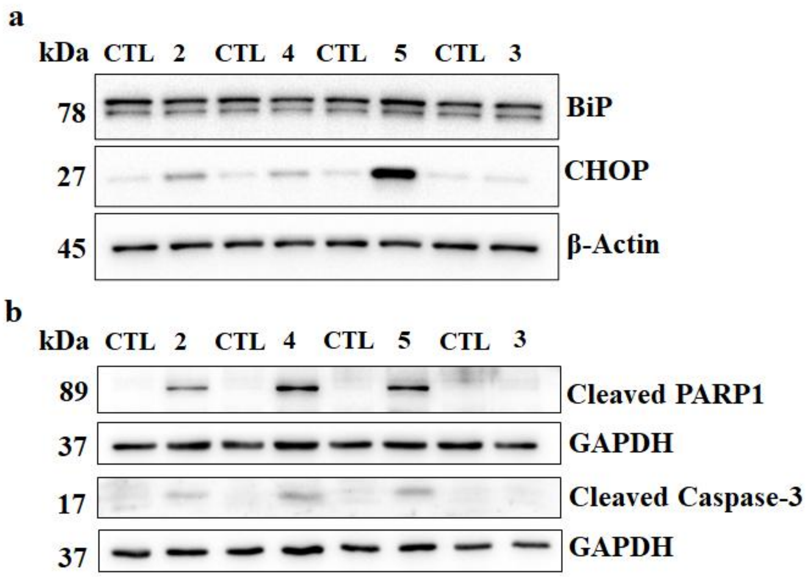

Figure S9. Western blot analysis of endoplasmic reticulum stress and apoptosis. Tested compounds were **2**, **3, 4,** and **5**. (a) Representative immunoblotting images of endoplasmic reticulum stress. NCI-H460 cells were treated with Os compounds (2 μM) for 24 h. (b) Representative immunoblotting images of apoptosis. NCI-H460 cells were treated with Os compounds (4 μM) for 24 h.

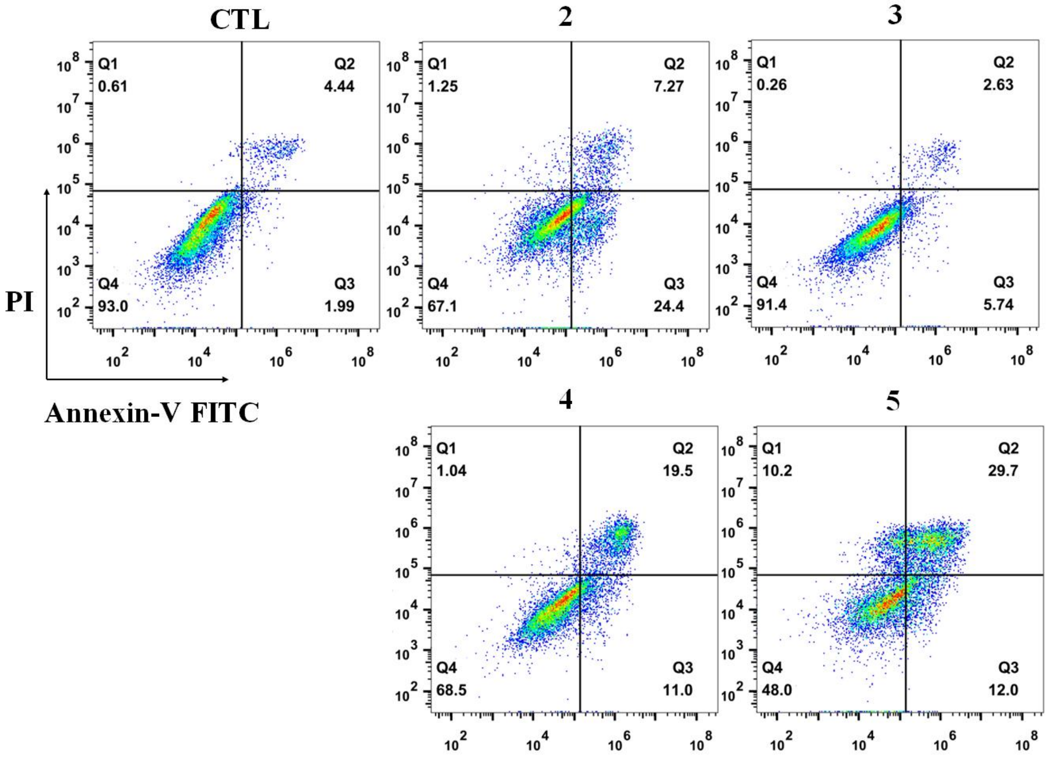

Figure S10. Flow cytometry results of apoptosis. NCI-H460 cells treated **2**, **3, 4** and **5**. (4 μM) for 24 h.

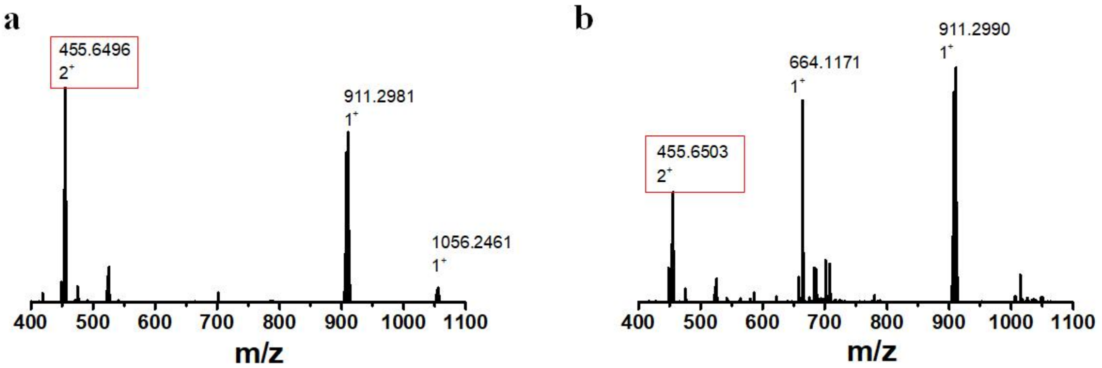

Figure S11. Oxidation of **Os(III)**. (a) HRMS spectrum of the reaction between the **Os(III)** (25 μM) and H_2O_2 (100 mM) for 3 hours. (b) HRMS spectrum of the reaction between the **Os(III)** (25 μM) and NAD^+ (500 μM) for 72 hours.

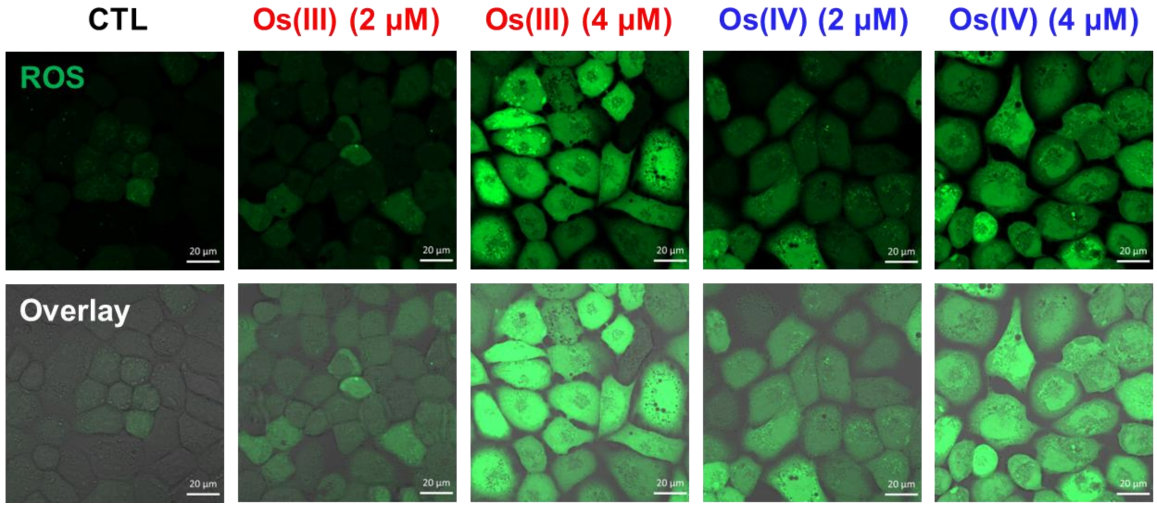

Figure S12. ROS detection in NCI-H460 cells. DCFH-DA as the fluorescence indicator. NCI-H460 cells were incubated with **Os(III)** or **Os(IV)** (2 and 4 μM) for 6 h. green fluorescence (λex = 488 nm, λem = 510–530 nm). Scale bars: 20 μm.

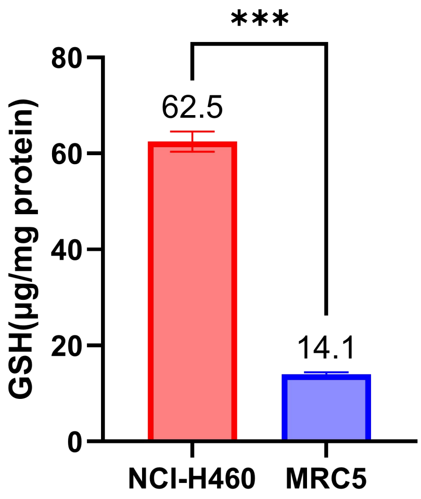

Figure S13. GSH contents in NCI-H460 and MRC5.

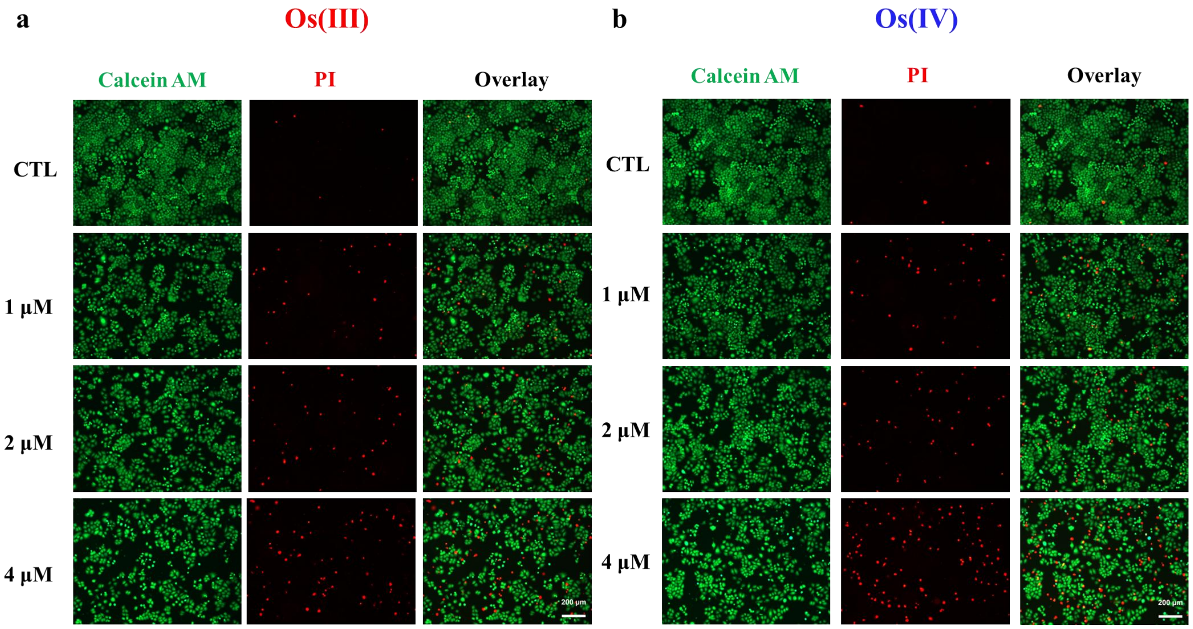

Figure S14. **Os(III)** or **Os(IV)** efficiently induce the death of cancer cells. NCI-H460 cells treated with **Os(III)** (a) or **Os(IV)** (b) at 1,2 and 4 µM for 24 h via live (green) and dead (red) staining. Scale bar: 200 µm.

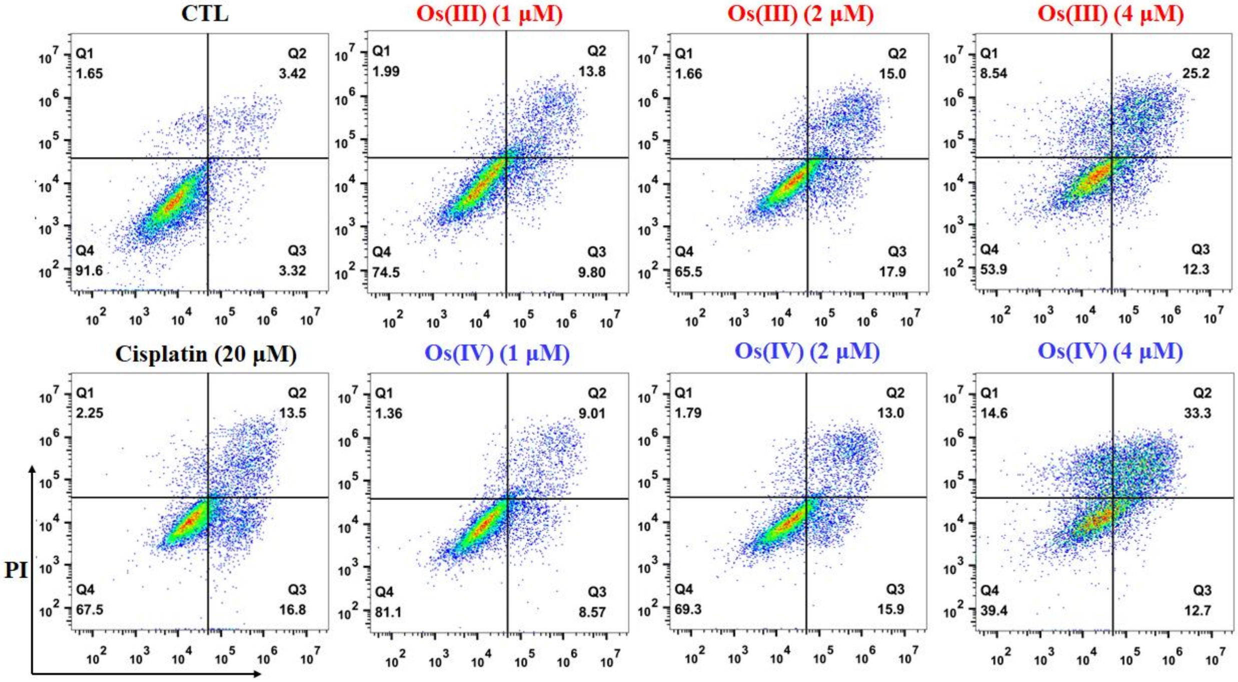

Figure S15. Induction of apoptosis of **Os(III)** or **Os(IV)**. NCI-H460 cells were treated with **Os(III)** or **Os(IV)** (1, 2, and 4 μM) for 24 h.

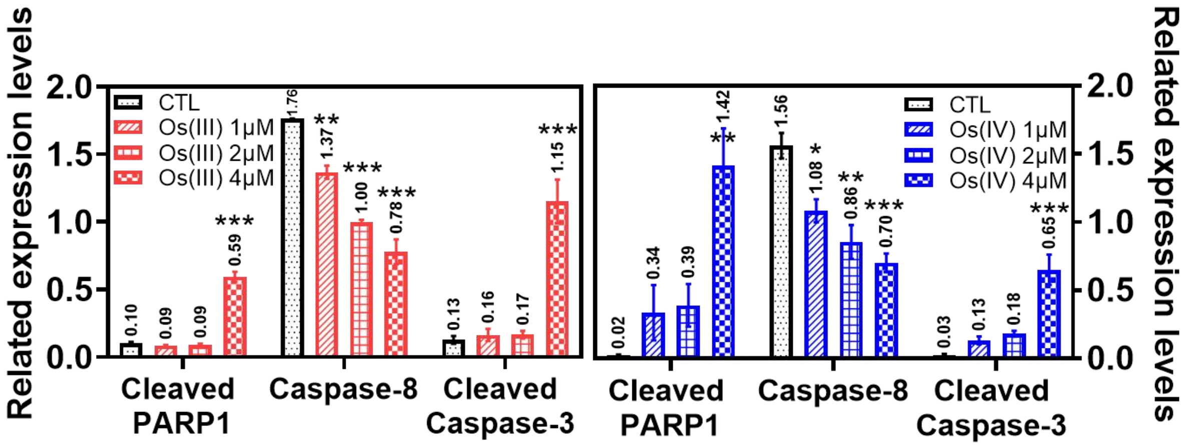

Figure S16. Gray value quantification of apoptosis-related proteins in cells exposed to **Os(III)/Os(IV)** (1, 2, and 4 μM) in **Figure 4e**.

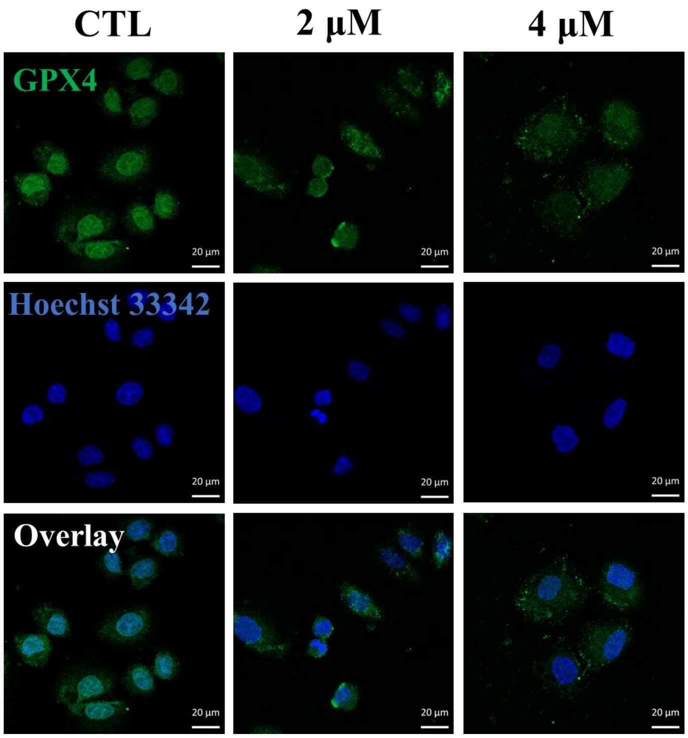

Figure S17. Immunofluorescence of GPX4 protein. NCI-H460 cells were incubated with **Os(IV)** (2 and 4 μM) for 24 h. Scale bar: 20 μm.

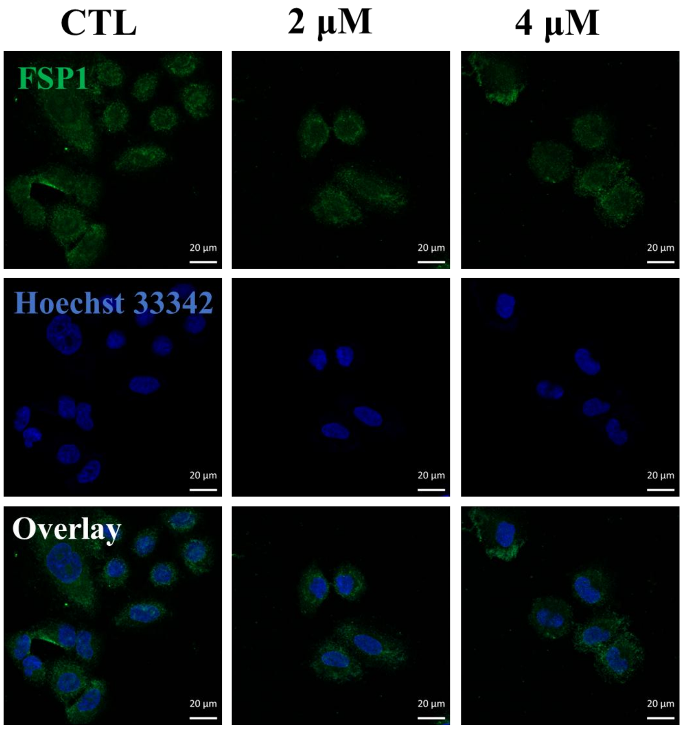

Figure S18. Immunofluorescence of FSP1 protein. NCI-H460 cells were incubated with **Os(IV)** (2 and 4 μM) for 24 h. Scale bar: 20 μm.

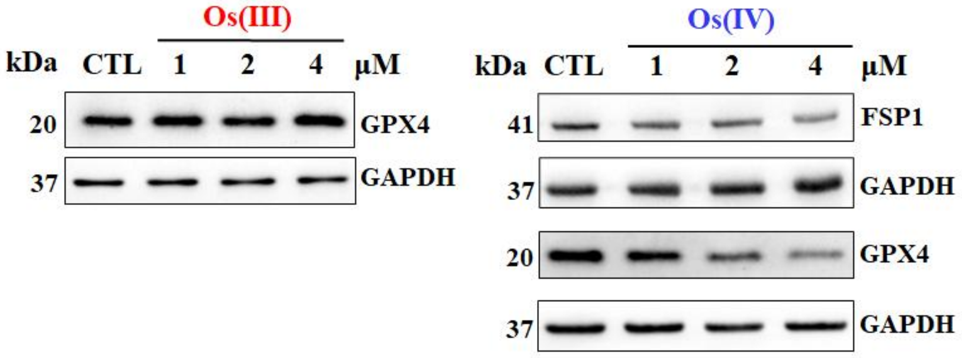

Figure S19. Representative western blot analyses of GPX4 and FSP1. NCI-H460 cells were incubated with **Os(IV)** (1, 2, and 4 µM) for 24 h.

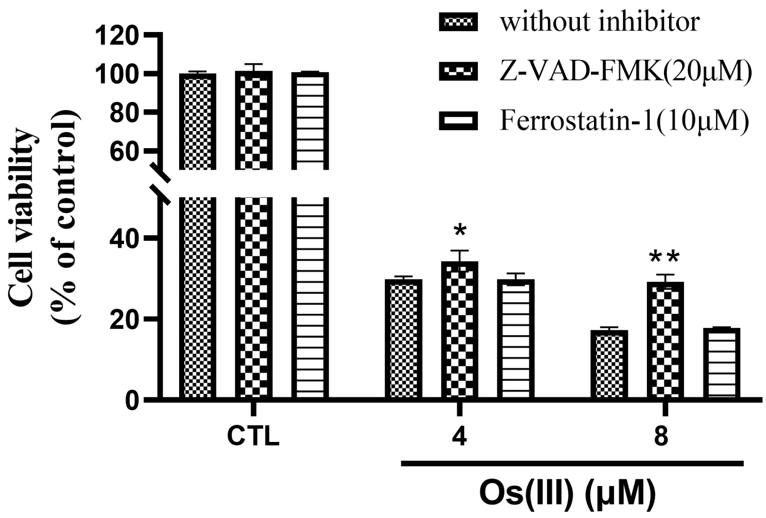

Figure S20. Cell viability in 24 h under incubation of Os(III) and inhibitors. **Os(III)**, **Os(III)** + Z-VAD-FMK (20 µM) and **Os(III)** + Ferrostatin-1 (10 µM) respectively. *$p<0.05$, **$p<0.01$.

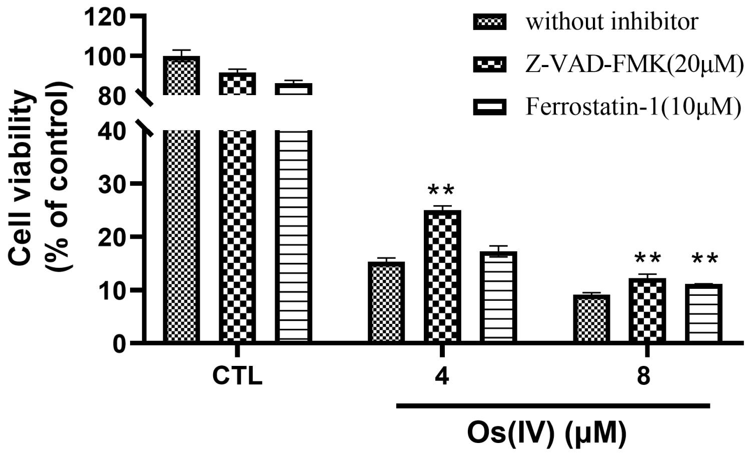

Figure S21. Cell viability in 24 h under incubation of Os(IV) and inhibitors. **Os(IV)**, **Os(IV)** + Z-VAD-FMK (20 μM), and **Os(IV)** + Ferrostatin-1 (10 μM) respectively. **p<0.01.

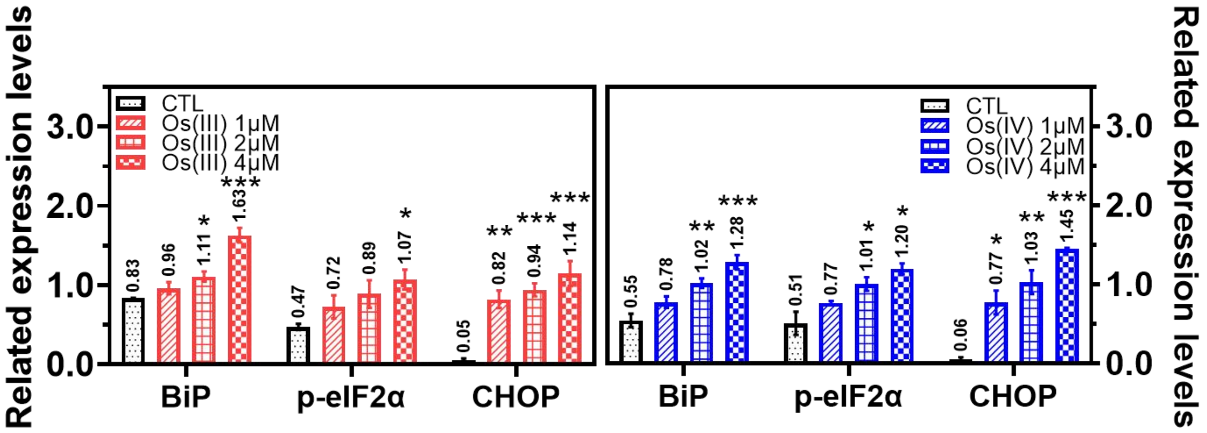

Figure S22. Gray value quantification of ER stress-related proteins in cells treated with **Os(III)/Os(IV)** (1, 2, and 4 μM) in **Figure 4h**.

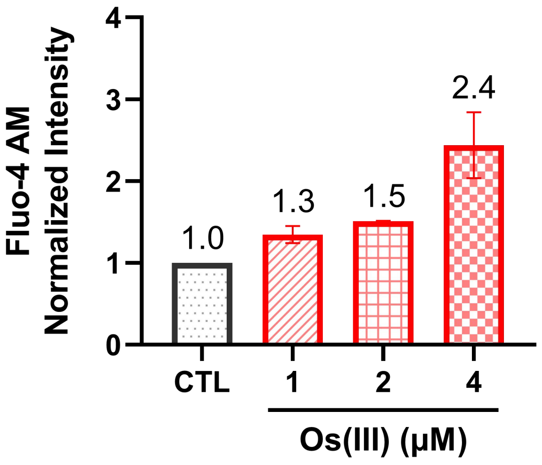

Figure S23. Flow cytometry results of a Ca^{2+} assay. NCI-H460 cells were incubated with **Os(III)** (1, 2, and 4 μM) for 24 h.

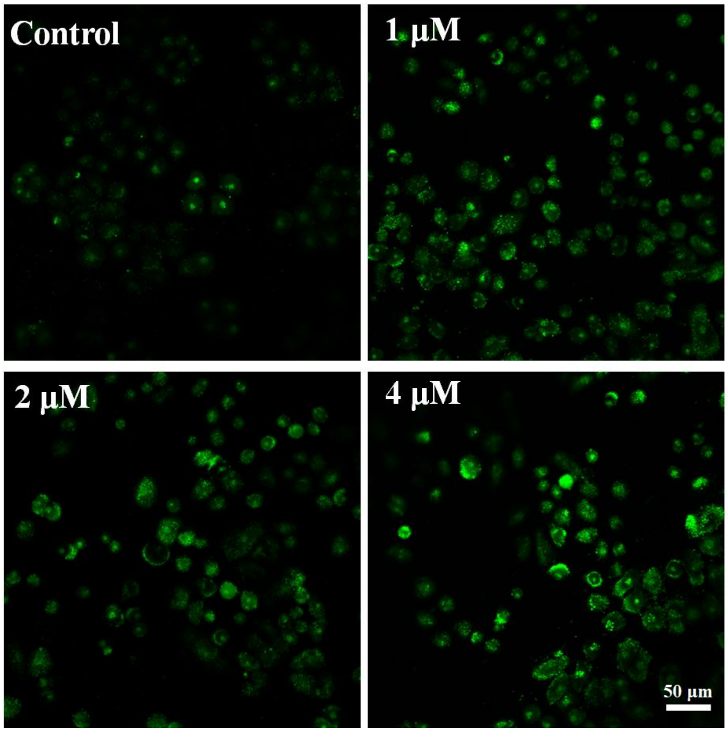

Figure S24. Confocal images of intracellular Ca^{2+} assays with Fluo-4 AM. NCI-H460 cells were treated with **Os(III)** (1, 2, and 4 μM) for 24 h, and then incubated with Fluo-4 AM for 0.5 h after being washed with PBS. Scale bar: 50 μm.

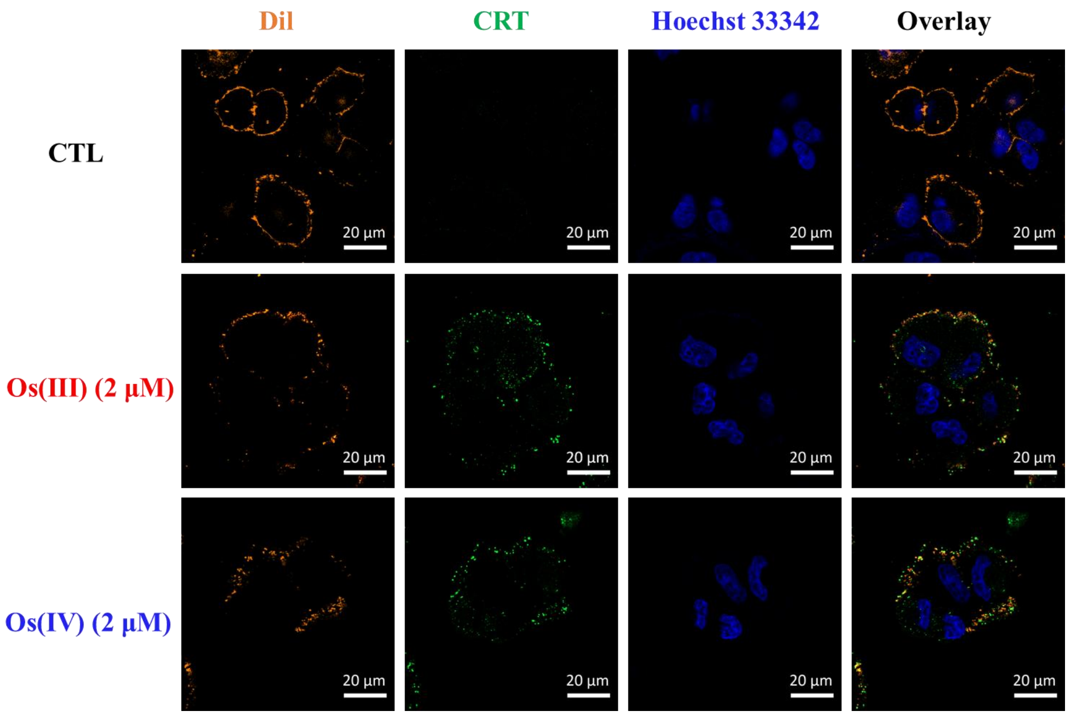

Figure S25. CRT translocation analysis. Confocal microscopy images of NCI-H460 cells incubated with the CRT-specific antibody and membrane-specific probe Dil (a lipophilic carbocyanine dye), treated with **Os(III)** or **Os(IV)** (2 μM) for 12 h. Scale bar: 20 μm.

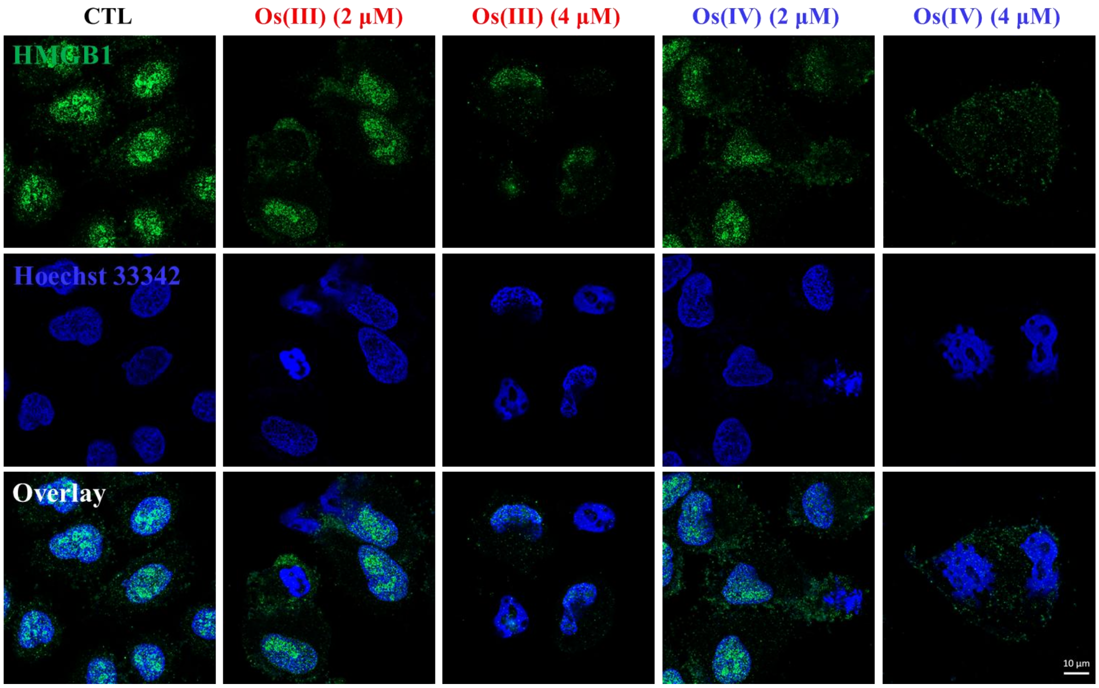

Figure S26. HMGB1 secretion analysis. Confocal microscopy images of NCI-H460 cells incubated with the HMGB1-specific antibody, treated with **Os(III)** or **Os(IV)** (2 and 4 μM). Scale bar: 10 μm.

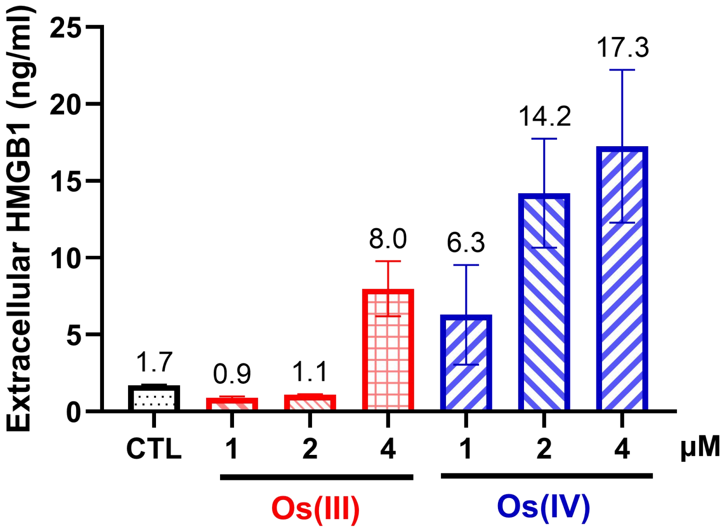

Figure S27. Release of HMGB1 in cell culture supernatant. NCI-H460 cells were incubated with **Os(III)** or **Os(IV)** (1, 2, and 4 μM) for 24 h.

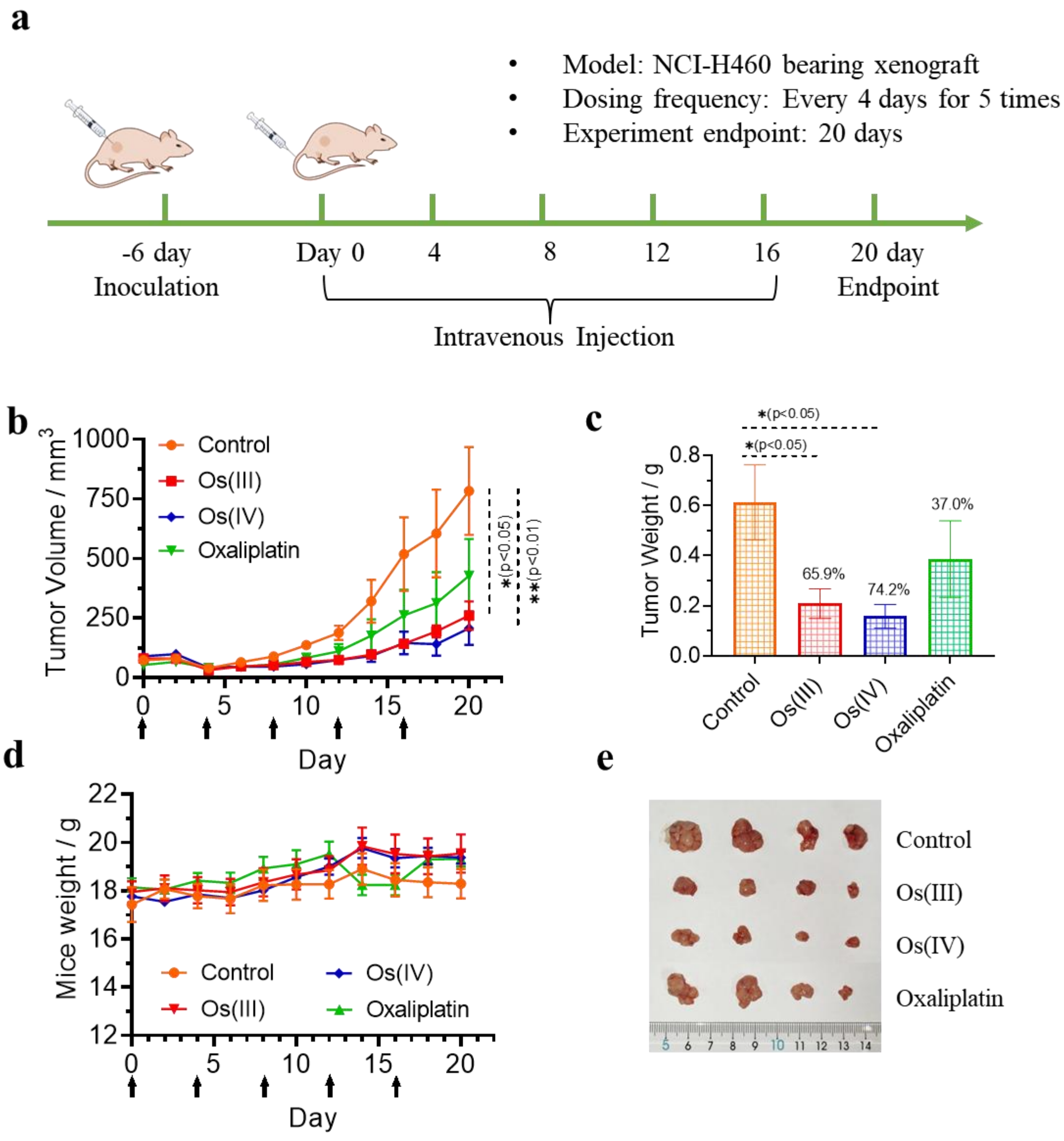

Figure S28. Antitumor effect of Os compounds on the NCI-H460 model. (a) Schematic illustration of the animal experimental design for assessing the antitumor effects of **Os(III)**/**Os(IV)** in a NCI-H460 xenograft model. Female BALB/c nude mice bearing NCI-H460 tumors were randomized into four groups (n = 4 per group): vehicle group (10% PET), **Os(III)** (5 mg/kg), **Os(IV)** (5 mg/kg), and Oxaliplatin (3 mg/kg). Treatments were administered via tail vein injection on days 0, 4, 8, 12, and 16; tumor volumes and body weights were monitored every 48 h. (b) Relative tumor volume changes over the course of treatment. (c) Tumor weights were measured at the end of the study. (d) Body weights of mice receiving treatment. (e) Isolated tumor tissues image at day 20 with different treatments.

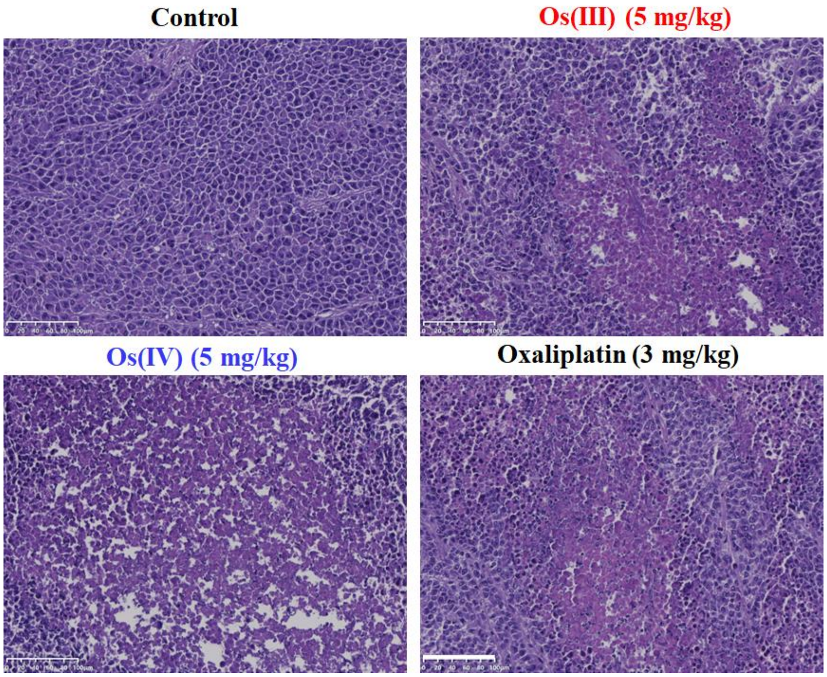

Figure S29. Hematoxylin−Eosin staining of tumors in different treatments on NCI-H460 model. (Scale bar: 100 μm).

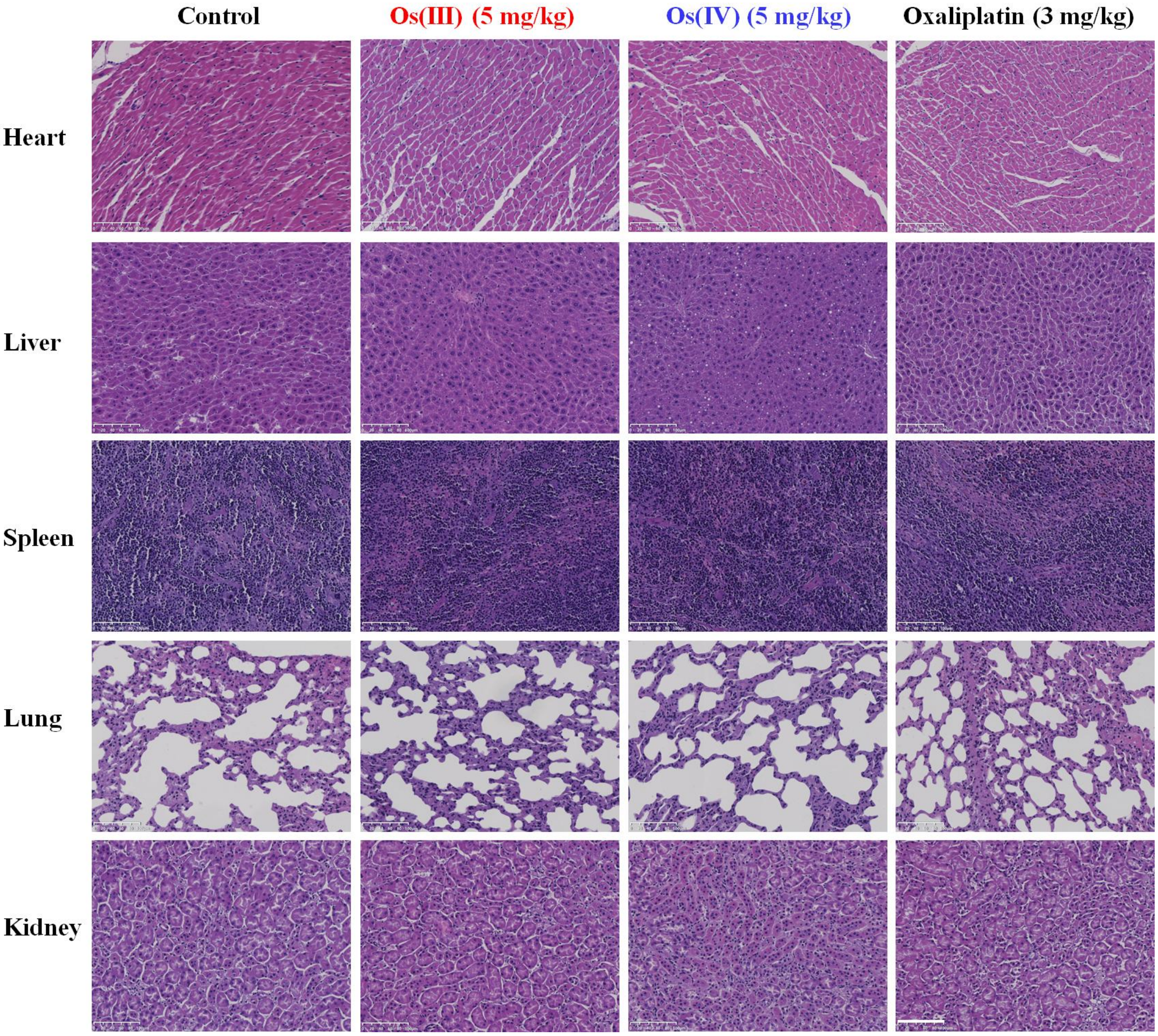

Figure S30. Hematoxylin−Eosin staining of major organs in different treatments on NCI-H460 model. (Scale bar: 100 μm).

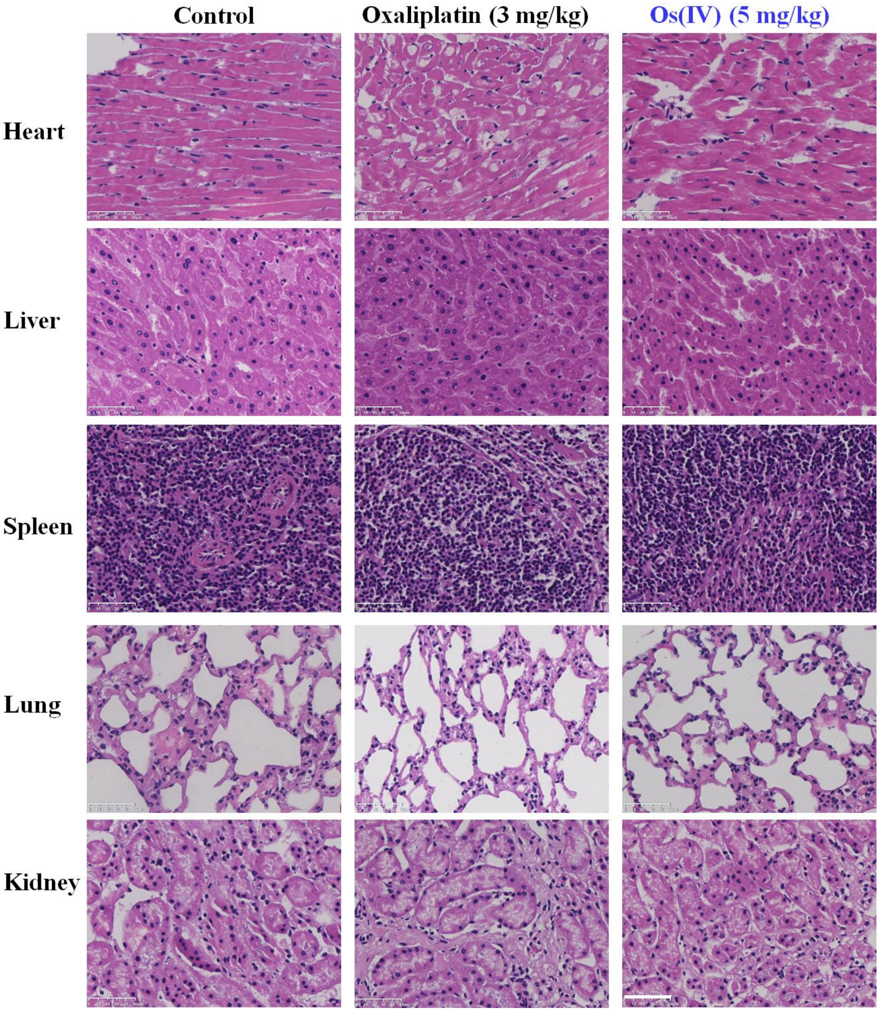

Figure S31. Hematoxylin−Eosin staining of major organs in different treatments on CT26 model. (Scale bar: 50 μm).

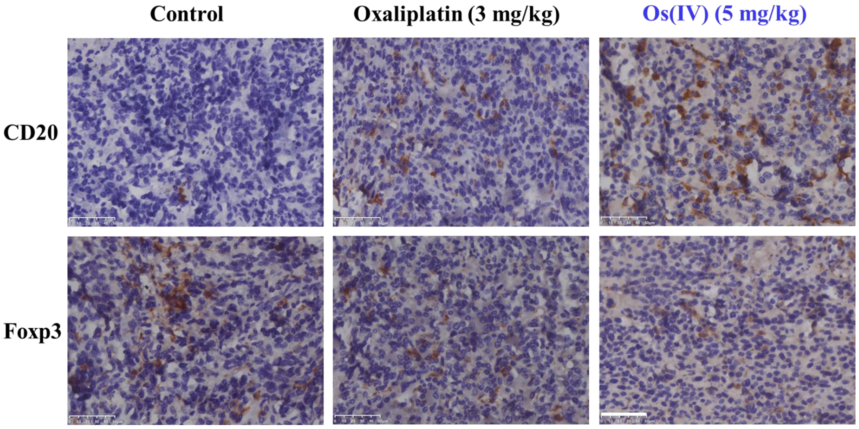

Figure S32. Immune response analysis of Os(IV) on CT26 model ($CD20^+$ B cells and Foxp3+ T cells). Immunohistochemistry of $CD20^+$ and $Foxp3^+$ T cells in tumour tissues of BALB/c mice treated with Os(IV) (Scale bar = 50 μm).

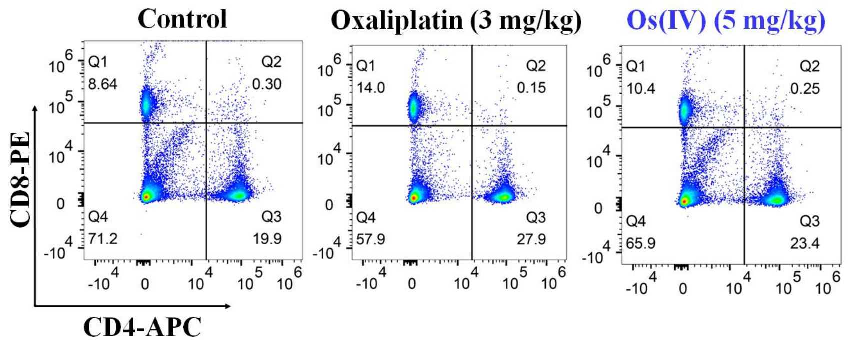

Figure S33. Immune response analysis of Os(IV) on CT26 model ($CD4^+$ and $CD8^+$ T cells in spleen). Flow cytometry analysis of $CD4^+$ and $CD8^+$ T cells in the spleen of BALB/c mice treated with different therapeutic agents.

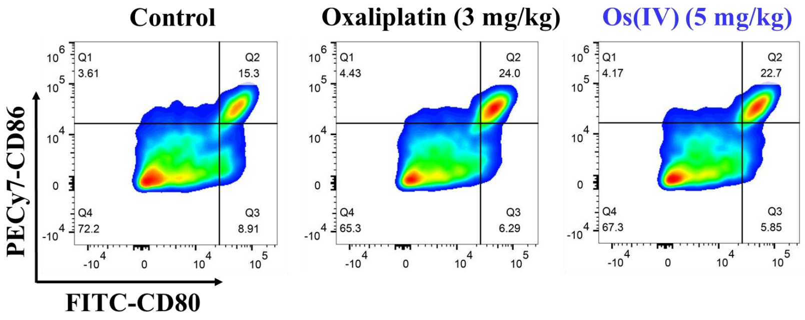

Figure S34. Immune response analysis of Os(IV) on CT26 model (DC cells in spleen). Flow cytometry analysis of CD80+ and CD86+ DC cells in the spleen of BALB/c mice treated with Os(IV).

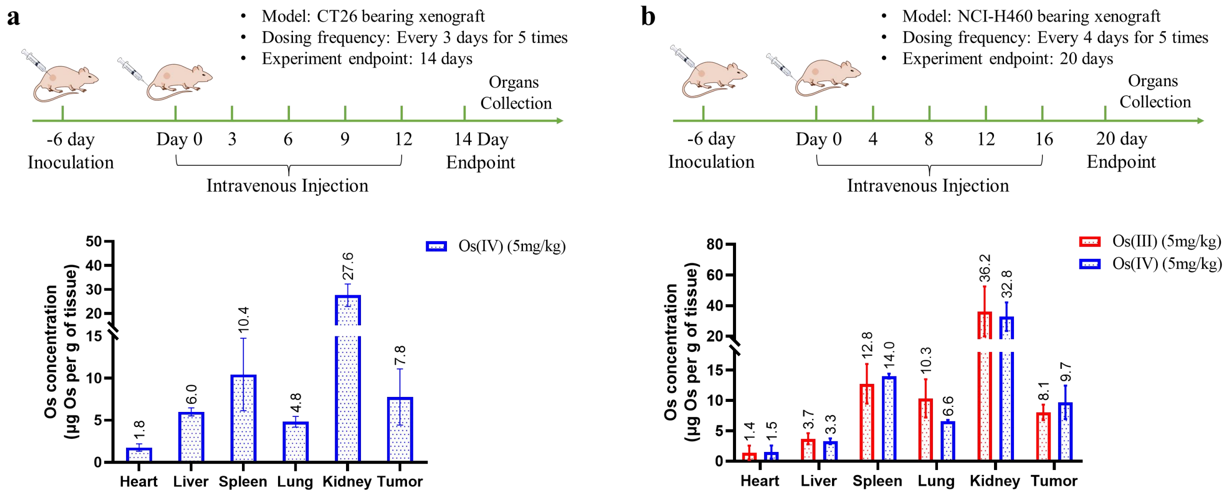

Figure S35. The concentration of Os was quantified in major organs (heart, liver, spleen, lung, kidney) and tumors of CT26 tumor-bearing mice(a) and NCI-H460 tumor-bearing mice(b).

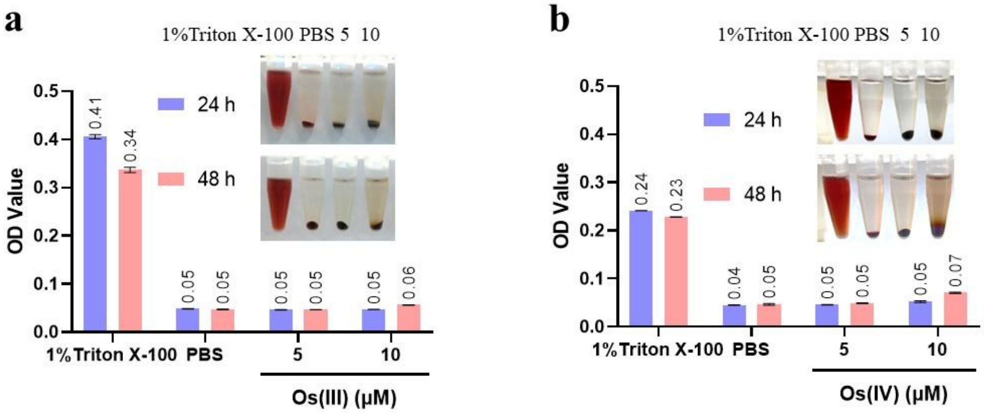

Figure S36. Hemolysis of red blood cells seen in the presence of various concentrations of Os(III) (a) and Os(IV) (b) at various indicated times. (Inset: A photograph showing the extent of hemolysis after centrifugation of the sample.)

Raw data of Western blot

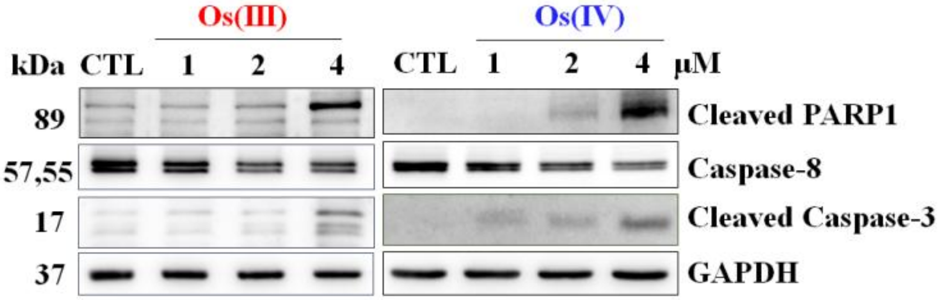

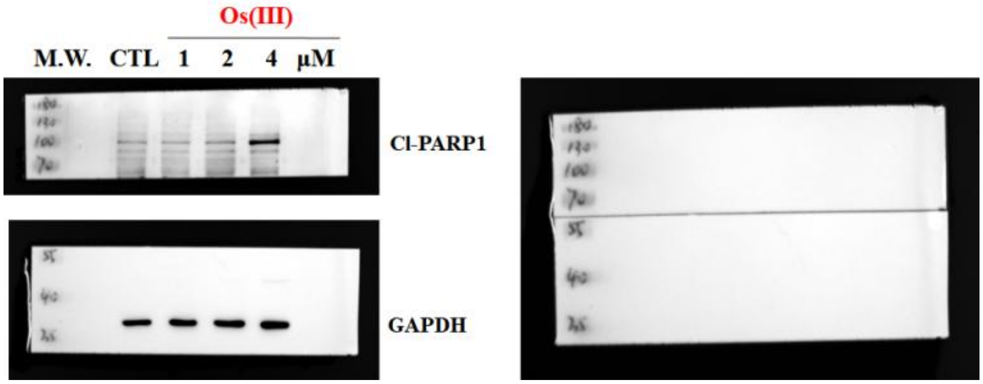

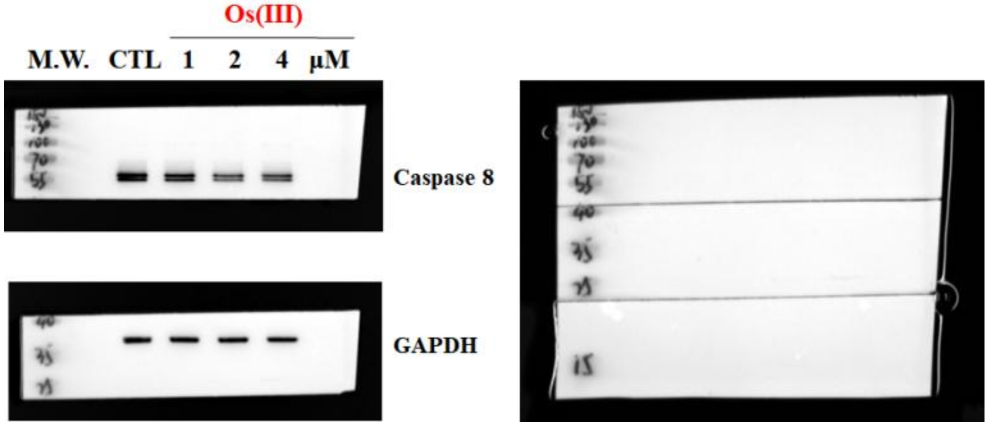

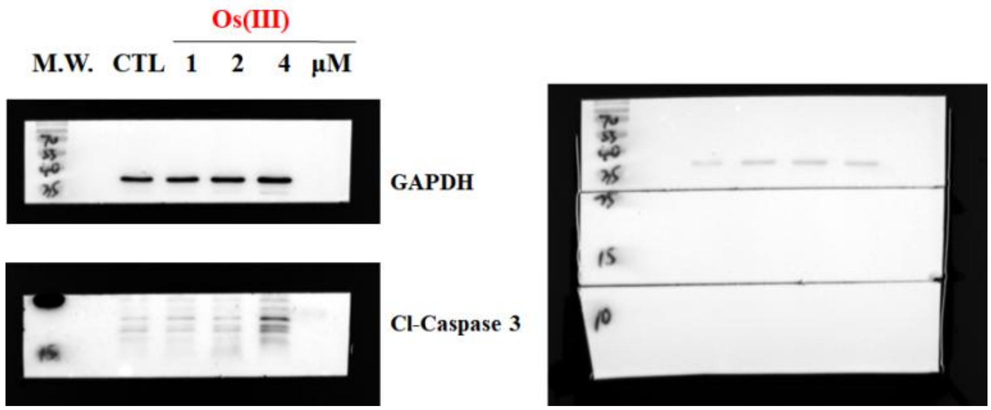
Os(III)
M.W. CTL 1 2 4 μM
GAPDH
Cl-Caspase 3

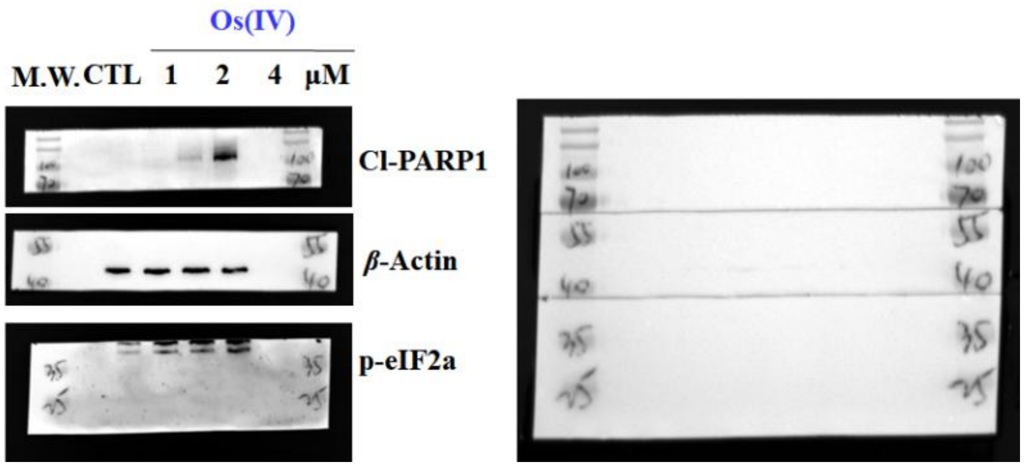
Os(IV)
M.W. CTL 1 2 4 μM
Cl-PARP1
β-Actin
p-eIF2a

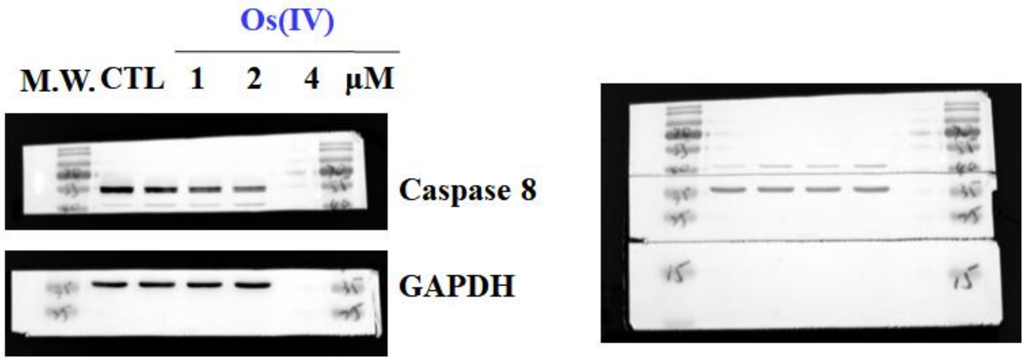
Os(IV)
M.W. CTL 1 2 4 μM
Caspase 8
GAPDH

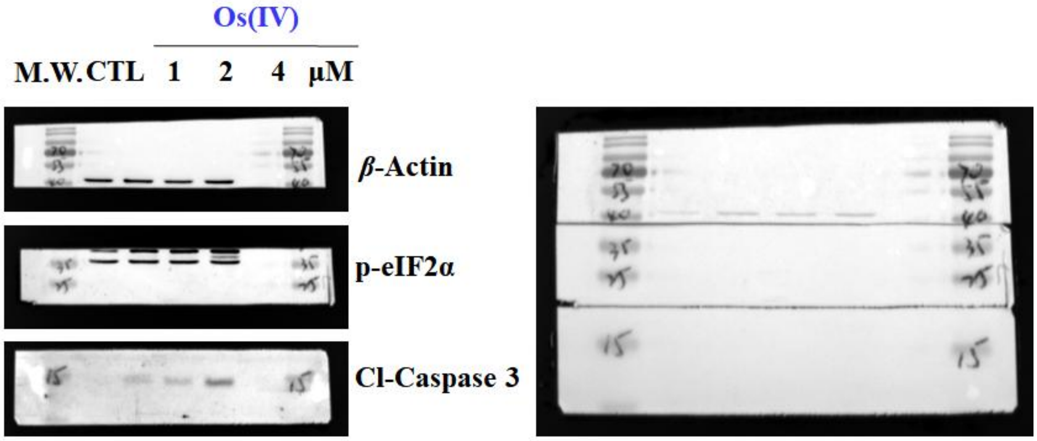
Os(IV)
M.W. CTL 1 2 4 μM
β-Actin
p-eIF2α
Cl-Caspase 3

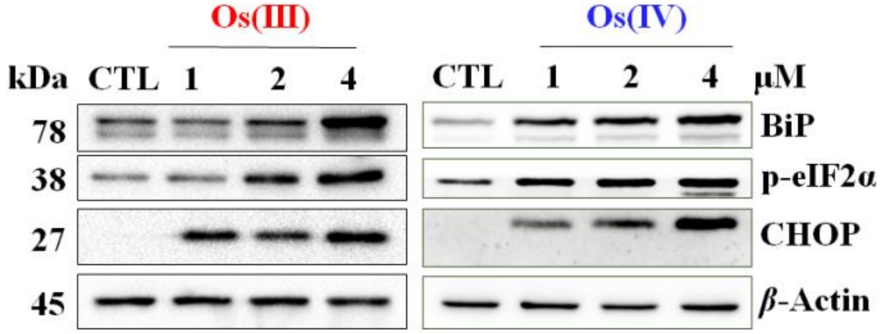
Os(III)
Os(IV)
kDa CTL 1 2 4
CTL 1 2 4 μM
78
38
27
45
BiP
p-eIF2α
CHOP
β-Actin

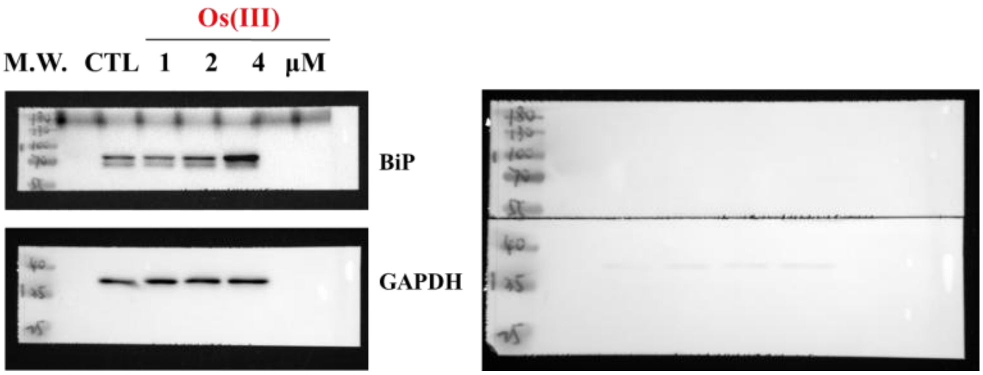
Os(III)
M.W. CTL 1 2 4 μM
BiP
GAPDH

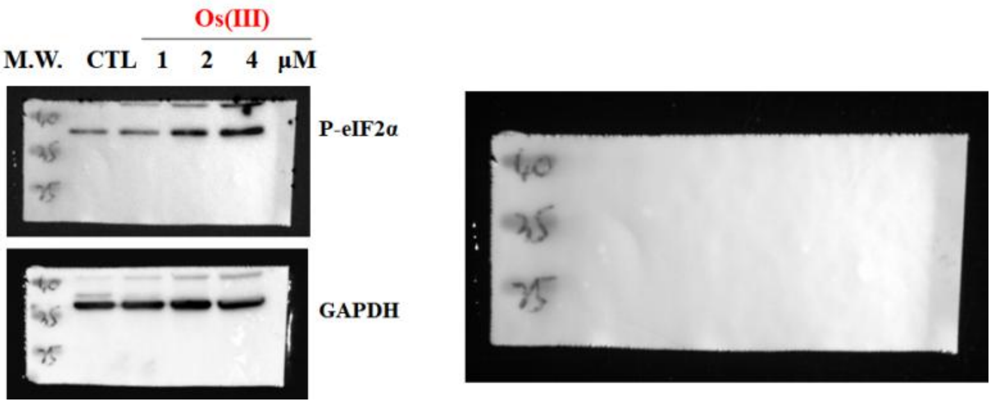
Os(III)
M.W. CTL 1 2 4 μM
P-eIF2α
GAPDH

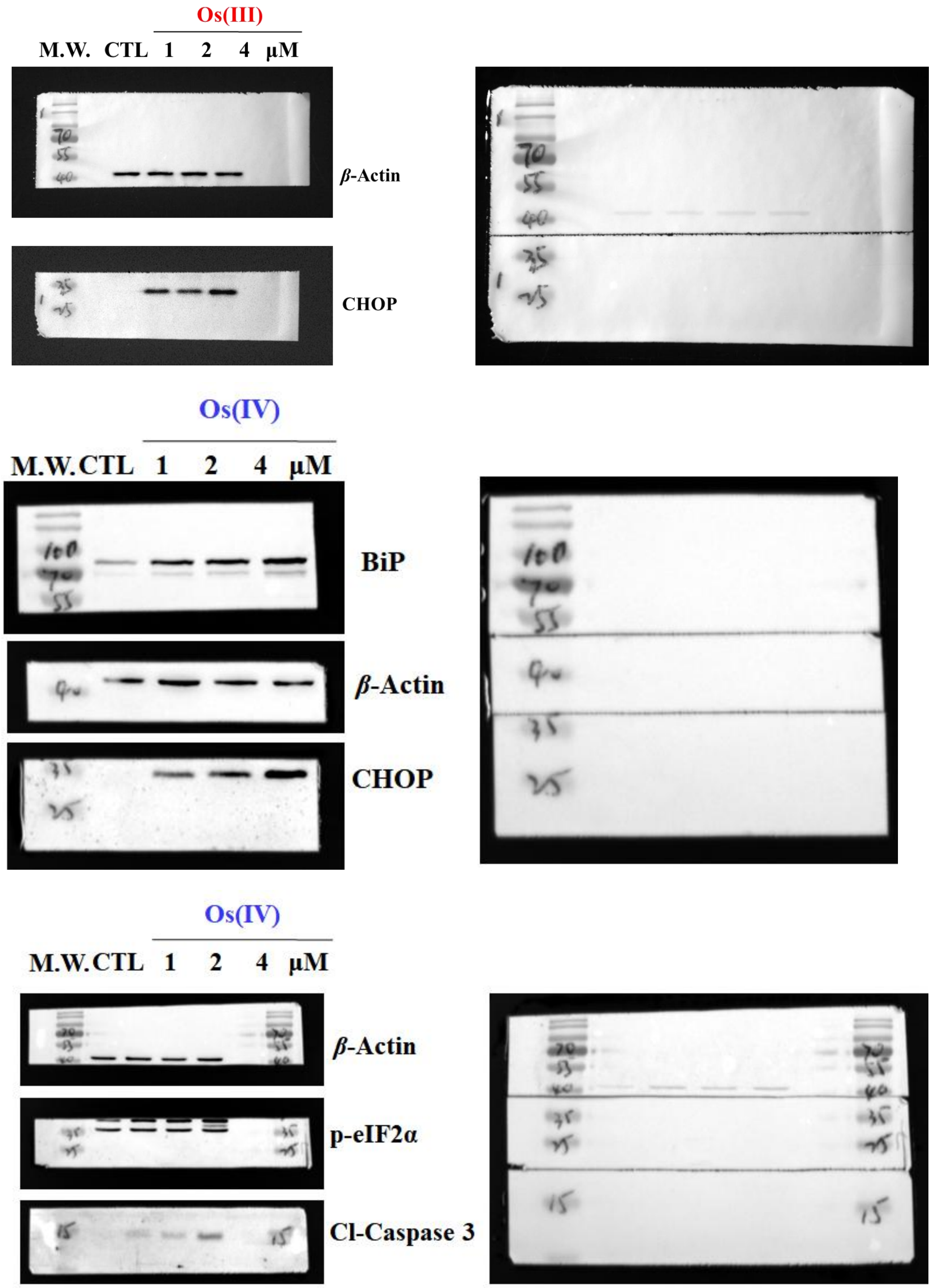

Figure S37. Unprocessed original membranes of Western blot shown in Figures 4e and 4h. M.W.: molecular weight ladder. According to the molecular weight of protein, the shown blots are cropped from different parts of the same gel, as explicitly shown by using clear delineation with dividing lines and white space.

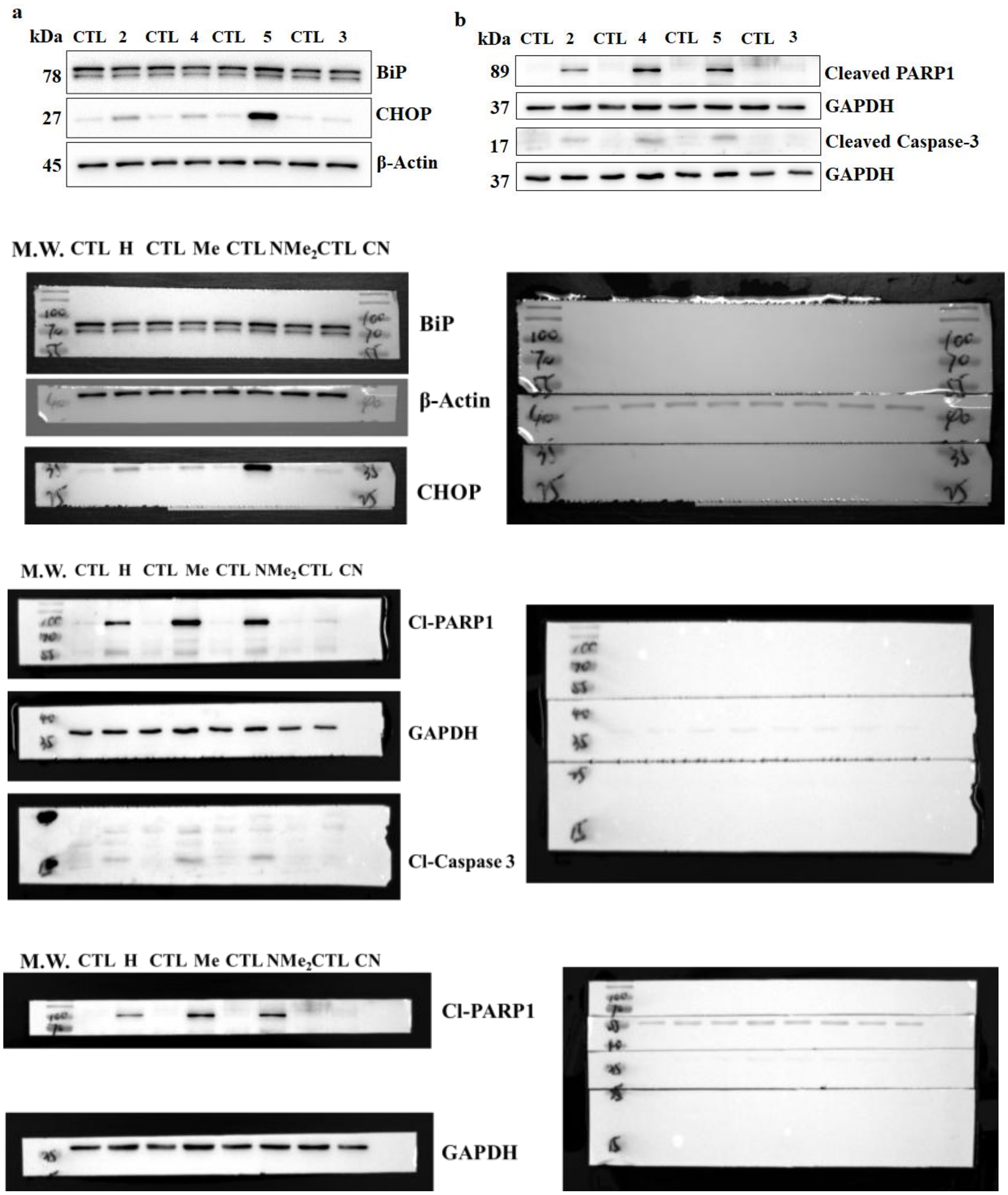

Figure S38. Unprocessed original membranes of Western blot shown in Figure S9. M.W.: molecular weight ladder. According to the molecular weight of protein, the shown blots are cropped from different parts of the same gel, as explicitly shown by using clear delineation with dividing lines and white space.

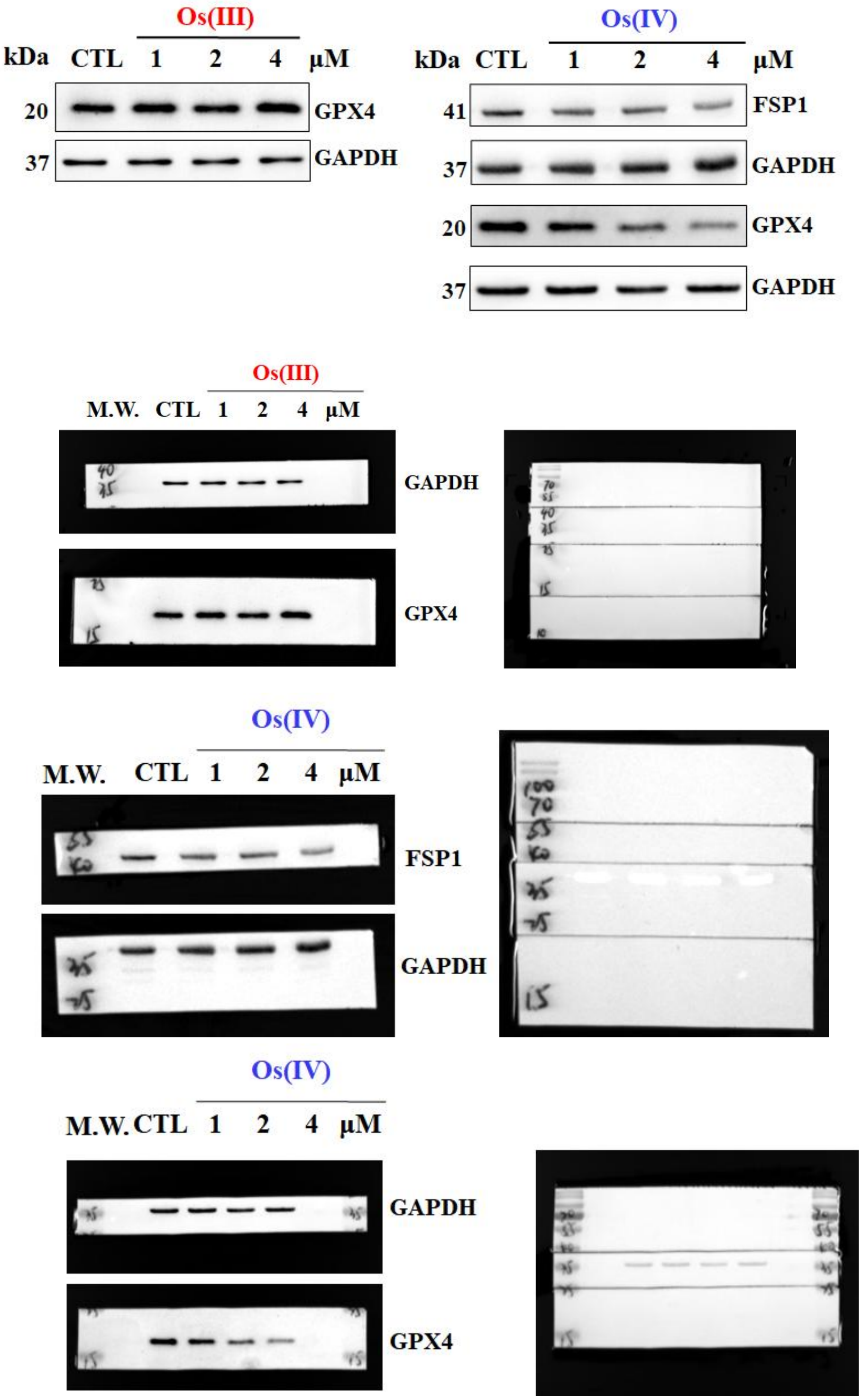

Figure S39. Unprocessed original membranes of Western blot shown in Figure S19. M.W.: molecular weight ladder. According to the molecular weight of protein, the shown blots are cropped from different parts of the same gel, as explicitly shown by using clear delineation with dividing lines and white space.
